# Supplementary material for: HIV-1 treatment timing shapes the human intestinal memory B-cell repertoire to commensal bacteria
Source: Nat Commun. 2023 Oct 10;14:6326. doi: 10.1038/s41467-023-42027-6 (PMC10564866; doi:10.1038/s41467-023-42027-6)
Supplement: Supplementary file 1 — Supplementary Information [file 41467_2023_42027_MOESM1_ESM.pdf]

Supplementary information

HIV-1 Treatment Timing Shapes the Human Intestinal Memory B-Cell Repertoire to Commensal Bacteria

Cyril Planchais[1], Luis M. Molinos-Albert[1,2], Pierre Rosenbaum[1], Thierry Hieu[1], Alexia Kanyavuz[3], Dominique Clermont[4], Thierry Prazuck[5], Laurent Lefrou[6], Jordan D. Dimitrov[3], Sophie Hüe[7], Laurent Hocqueloux[5], Hugo Mouquet[1, 8]

.

[1]Humoral Immunology Unit, Institut Pasteur, Université Paris Cité, INSERM U1222, F-75015 Paris, France
[2]Current address: ISGlobal, Hospital Clínic-Universitat de Barcelona, 08036 Barcelona, Spain
[3]Centre de Recherche des Cordeliers, INSERM, Sorbonne Université, Université de Paris, 75006 Paris, France
[4]Collection of the Institut Pasteur, Institut Pasteur, Université Paris Cité, 75015 Paris, France
[5]Service des Maladies Infectieuses et Tropicales, CHR d'Orléans-La Source, 45067 Orléans, France
[6]Service d'Hépato-Gastro-Entérologie, CHR d'Orléans-La Source, 45067 Orléans, France
[7]INSERM U955 - Équipe 16, Université Paris-Est Créteil, Faculté de Médecine, 94000 Créteil, France

Supplementary tables 1 and 2

Supplementary figures 1 - 5

Supplementary table 1. Clinical and immunovirological characteristics of eART and lART donors

	#	Gender	Age at inclusion (years)	Duration with HIV infection (years)	Time on cART (years)	ART initiation (estimated day post-HIV diagnosis)	Fiebig stage at the start of ART	Duration with PVL<50 copies/ml (years)	nadir CD4 / mm^3	CD4$^+$ T cells (%)	CD8$^+$ T cells (%)	CD4+ T cells (cells/mm^3)	CD8+ T cells (cells/mm^3)	CD4 / CD8 ratio
eART	E1	F	36	3.2	3.2	10	III	3.1	854	49.8	35.1	1768.4	1246	1.4
	E2	M	65	7.5	7.5	7	IV	6.9	369.3	40.4	23.2	833	478	1.7
	E3	F	48	3.5	3.5	8	V	2.9	374.6	56	25	772.8	345	2.2
	E4	F	36	3.4	3.4	26	V	2.9	521.4	49	25	1353	690	2.0
	E5	M	53	5.1	5.1	2	IV	4.7	1253	54	19	1863	647	2.9
	E6	F	41	19.4	19.4	0	IV	1.5	524.3	43	43	864	868	1.0
	E7	M	38	1.4	1.4	7	III	1.3	608.8	42	36	1125	954	1.2
	E8	M	55	11.6	11.6	3	III	4.0	815	41	22	1060	579	1.8
	E9	M	69	5.7	5.7	1	V	4.9	401.5	38.6	36.4	475.6	449	1.1
	E10	M	41	6.1	6.1	21	V	5.9	570	44	21	947	462	2.0
	E11	M	31	9.9	9.9	6	V	9.2	405	34.2	46.4	731.9	993	0.7
	E12	M	70	10.6	10.5	27	V	0.9	500.1	41	41	872	885	1.0
	E13	M	46	11.5	11.5	20	V	3.9	368.5	43	26	668	403	1.7
	E14	M	44	8.4	8.3	15	V	7.4	450	41	42	1051	1070	1.0
	E15	M	53	10.6	10.6	26	V	9.2	664	43	42	937	913	1.0
	E16	F	51	17.9	17.7	76	V	0.3	496.3	51.4	27.7	1459.2	787	1.9
	E17	M	63	2.1	2.1	17	V	1.2	249	36	26	519	373	1.4
	E18	M	47	3.2	3.2	16	IV	2.7	355.7	39	35	538	483	1.1
	E19	M	40	3.5	3.4	18	IV	2.0	262.8	38	37	831	818	1.0
	E20	M	63	12.9	12.8	35	V	3.8	445.6	43	34	676	534	1.3
	E21	M	55	2.4	2.3	18	IV	1.5	214.9	44	33	461	342	1.3
	E22	M	35	3.6	3.6	6	III	3.2	582.8	40	29	1148	832	1.4
	E23	M	43	9.7	9.6	20	IV	8.0	723.4	50	29	1094	646	1.7
	E24	M	51	14.5	14.4	15	V	8.8	329	37	40	952	1050	0.9
	E25	F	79	19.5	19.3	54	V	7.5	407.9	48	20	1067	452	2.4
	E26	M	40	3.2	3.2	11	IV	2.8	627.4	47	30	1374	878	1.6
	E27	M	68	10.5	10.5	12	IV	8.7	390.6	24	32	611	806	0.8
	E28	M	32	3.2	3.2	9	IV	3.1	442.5	50	30	1004	597	1.7
	E29	M	57	3.8	3.7	40	V	3.6	732	38	22	836	470	1.8
	E30	M	45	13.1	13.0	10	V	3.5	320	39.9	48	551	663	0.8
	E31	M	53	16.7	16.7	9	V	16.0	402.1	33	28	861	731	1.2
	E32	M	48	23.0	22.8	52	V	11.2	320.6	36	40	527	587	0.9
	E33	M	56	9.8	9.7	8	IV	8.8	392	30	40	572	750	0.8
	E34	M	49	2.2	2.2	18	V	1.9	283.1	40	42	708	742	1.0
	E35	M	28	1.7	1.7	0	V	1.3	334.4	37.7	38.4	941.4	960	1.0
	E36	M	36	2.5	2.5	3	V	2.2	589.4	33	37	1101	1216	0.9
	E37	M	35	3.0	3.0	5	II	1.7	293.6	25	31	493	612	0.8
	E38	F	57	10.0	9.9	22	V	9.7	835	52	34	2309	1501	1.5
lART	L1	M	58	11.6	8.2	1242	VI	4.4	205.2	36.2	42.7	925.6	1092	0.8
	L2	F	69	21.5	21.2	105	VI	10.3	284.8	36	33	792	726	1.1
	L3	M	50	22.4	21.2	454	VI	3.9	63.9	29	38	765.6	1003	0.8
	L4	F	80	19.1	19.0	21	VI	10.3	6.3	34.2	38.8	221	251	0.9
	L5	M	52	22.8	9.9	1887	VI	6.7	125	19.5	32	377	531	0.7
	L6	F	34	13.0	12.9	28	VI	3.7	227.8	35.7	32.9	479	442	1.1
	L7	F	54	12.8	12.8	5	VI	4.8	231	30.3	20.4	1564.1	1052	1.5
	L8	F	53	5.3	3.2	775	VI	1.5	341	46	32.2	915.4	640	1.4
	L9	F	52	8.6	5.3	1217	VI	5.0	307	43.2	24.5	709.3	403	1.8
	L10	M	43	10.7	10.1	232	VI	6.3	306	49	28.9	1120	665	1.7
	L11	F	38	10.8	10.7	8	VI	0.0	251.1	35.7	28.4	643	512	1.3
	L12	M	72	10.6	10.5	51	VI	6.5	149.1	30.5	26.6	597.2	521	1.1
	L13	F	57	3.5	3.4	12	VI	3.1	343	40.1	50	978	1219	0.8
	L14	M	46	19.5	17.7	660	VI	6.6	287.3	42.3	38	933	840	1.1
	L15	M	50	0.3	0.2	29	VI	0.0	29	12.7	78.3	59.2	365	0.2
	L16	M	50	14.5	14.5	0	VI	2.2	169.2	23.5	35.2	475	713	0.7
	L17	F	69	10.8	9.2	567	VI	8.9	154	37.2	41.9	762	866	0.9
	L18	M	73	9.1	9.1	4	VI	8.5	164	32.3	26.7	501.6	415	1.2
	L19	M	38	0.3	0.2	26	VI	0.0	69	11.7	61.8	88	465	0.2
	L20	M	52	16.1	16.0	31	VI	8.2	76	27.3	52.9	494	957	0.5
	L21	M	69	31.6	11.6	7300	VI	0.5	147	16.5	66.2	269.6	1082	0.2
	L22	M	63	3.1	3.1	28	VI	2.7	616	43	31.6	1127	828	1.4
	L23	M	39	13.1	11.6	548	VI	4.6	479.5	37.2	29.3	702.7	554	1.3
	L24	M	60	16.7	16.7	19	VI	1.0	6	8.8	17.9	446.2	905	0.5
	L25	M	41	2.7	2.2	197	VI	1.8	665	35	34.3	1386	1356	1.0
	L26	F	42	4.9	4.9	0	VI	4.5	398	33.9	51.5	892	1355	0.7
	L27	F	26	25.9	20.0	2148	VI	0.0	11.1	38.9	56.3	26	620	0.0
	L28	F	47	13.9	13.9	17	VI	1.8	290.8	42.6	30.2	759	539	1.4
	L29	F	47	5.6	5.6	18	VI	5.3	167.4	39.6	41.6	379	398	1.0
	L30	F	23	1.6	0.6	376	VI	0.0	505	35.6	35	934.1	918	1.0
	L31	M	64	17.4	17.3	32	VI	1.3	127.7	39.5	43.7	421	465	0.9
	L32	F	57	0.4	0.2	83	VI	0.0	172	13.4	68.3	235	1201	0.2
	L33	M	49	4.6	4.6	10	VI	0.5	229	38.9	25.9	1020	679	1.5
	L34	F	27	8.9	6.6	828	VI	0.0	82	16.5	52.2	224	707	0.3
	L35	F	64	13.1	12.9	68	VI	12.6	214.3	28.3	38.6	496	676	0.7
	L36	F	31	5.6	5.6	15	VI	5.0	300.8	31.8	42.2	864.3	1146	0.8
	L37	F	54	20.9	19.8	402	VI	12.5	212.4	29.9	36.3	676.9	821	0.8
	L38	F	45	3.5	3.5	0	VI	2.5	459.3	20.4	47.1	663	773	0.9
	L39	F	33	15.1	3.1	4395	VI	0.0	131	20.4	47.1	688.1	1588	0.4
	L40	M	45	19.7	3.0	6097	VI	2.7	213	37	40.2	464.7	505	0.9

Table S2. Immunoglobulin gene repertoire and reactivity of intestinal IgA+ and IgG+ memory B-cell antibodies from HIV-1-infected donors under ART.

	Gene repertoire																					Reactivity									
	Heavy chain											Light chain											HEp-2		Commensal bacteria						
mAb #	VH	DH	JH	Subtype	CDRH3	Length	(+)	(-)	GRAVY	n SM	% SM	κ/λ	Vκ/λ	Jκ/λ	CDRL3	Length	(+)	(-)	GRAVY	n SM	% SM	PolyR	ELISA	IFA	E. coli	E. faecalis	M. morganii	E. cloacae	P. corporis	B. subtilis	C. difficile
E1-102	4-39	5-24	5	γ1	QRWLQLIDQ	9	1	1	-0.81	24	7.2	λ	1-44	3	APWDDSLNGFI	11	0	2	-0.12	4	1.3	/	/	/	/	/	/	/	/	/	/
E1-105	1-69/1-69D	3-10/3-9	5	α1	STDFDRFDPDWFDP	14	1	5	-1.37	20	6.0	κ	3-20	1	QQYGYSPRT	9	1	0	-1.96	12	4.4	/	/	/	/	/	/	/	/	/	/
E1-108	3-21	3-10	4	γ1	DLTPIRGITSAVGY	14	1	1	0.35	5	1.5	λ	2-8	3	SSYAGSVV	8	0	0	0.76	3	1.0	X	/	/	/	/	/	X	X	/	X
E1-110	3-48	2-21	2	α1	TLWGGDWYFDL	11	0	2	-0.11	19	5.9	λ	2-14	1	SSSTTDTTLLCV	12	0	1	0.47	12	3.9	X	/	/	/	/	/	/	/	/	/
E1-114	1-2	3-10	5	α1	AIPPPFVTIIRGALGRFDP	19	2	1	0.54	11	3.2	λ	3-21	3	QVWDSISDRWV	11	1	2	-0.50	8	2.8	/	/	/	/	/	/	/	/	/	/
E1-115	4-38-2	3-22	5	γ2	RYYDSTGYYANYFDP	15	1	2	-1.36	8	2.4	κ	1D-39/1-39	1	QQSYSTWWT	9	0	0	-1.46	8	3.1	X	X	/	X	X	X	X	X	X	X
E1-117	3-23	2-2/2-8/3-16	5	α1	DPKYTTTWYDGDWFDP	16	1	4	-1.58	27	8.0	κ	3-11	4	QQRSDWPRLT	10	2	1	-1.97	14	4.9	/	/	/	/	/	/	/	/	/	/
E1-118	1-46	2-15/3-3	4	α1	DNHKWSFDY	9	2	2	-1.98	10	3.1	λ	2-14	3	SSYTSRDSWD	10	1	2	-1.76	9	3.0	/	/	/	/	/	/	/	/	/	/
E1-123	4-59	1-1/1-7	4	γ1	NWNLSPLDY	9	0	1	-0.83	17	5.6	κ	1D-39/1-39	4	QQSYILPLT	9	0	0	0.08	20	7.7	/	/	/	/	/	/	/	/	/	/
E1-124	3-23	3-3	4	α1	VGTLRFLEWRFDY	13	2	2	-0.15	17	5.2	κ	4-1	1	QQHYSTPPT	9	1	0	-1.88	10	3.5	/	/	/	/	/	/	/	/	/	/
E1-131	3-30/3-30-3	6-13	4	α1	GYSSTYPSDFAY	12	0	1	-0.66	7	2.1	λ	6-57	3	QSYHSSNHWV	10	2	0	-1.38	9	1.0	/	/	/	/	/	/	/	/	/	/
E1-137	4-4	1-26/3-10	5	γ1	DRELSS	6	1	2	-1.55	16	5.2	κ	3-11	1	QQRSNWPPWT	10	1	0	-2.15	11	4.1	/	/	/	/	X	X	X	X	X	X
E1-139	3-7	3-3	5	α1	VMEPLRFLEYLRGLNWLDA	19	2	3	0.13	31	8.9	κ	1-6	1	LQDYNYPWT	9	0	1	-1.39	15	5.7	X	X	/	/	X	X	X	X	X	X
E1-140	4-34	2-15	6	γ1	KECGGSCYSTSYYYYYGMDV	20	1	2	-0.60	24	6.9	κ	2D-28/2-28	4	MQALQSPLT	9	0	0	0.13	11	3.6	/	/	/	/	/	/	/	/	/	/
E1-147	4-59	2-8	5	α1	GDGVSYHNRFDP	12	2	2	-1.31	33	10.2	λ	6-57	3	QSYDDSNHVV	10	1	2	-1.17	14	2.7	/	/	/	/	/	/	/	/	/	/
E1-148	1-58	3-16	6	α1	DKERYSYVSYYVMDV	15	2	3	-0.75	23	6.3	κ	1D-39/1-39	2	QQSFSTPYT	9	0	0	-1.12	20	7.3	/	/	/	/	/	/	/	/	/	/
E1-152	4-61	2-15/2-21	1	α2	ARPGVVGPSEH	11	2	1	-0.53	27	8.2	κ	1-5	2	QQYSSSPYT	9	0	0	-1.59	11	4.2	/	/	/	/	/	/	/	/	/	/
E1-156	3-30/3-30-3	3-16	5	γ2	DNYAYTFGS	9	0	1	-0.77	25	7.9	λ	2-23	3	CSFAGSGTWV	10	0	0	0.73	19	6.3	/	/	/	/	/	/	/	/	/	/
E1-156	4-59	3-16/7-27	4	α2	HGDGGKFDY	9	2	2	-1.53	15	4.8	κ	1-5	5	QQYNSYPIT	9	0	0	-1.30	10	4.0	X	/	/	X	X	X	X	X	X	X
E1-157	4-39	3-10	4	γ1	HPSTHYGAFPQRYPHFDY	18	4	1	-1.35	7	2.0	λ	1-47	3	AAWDDSLSGL	10	0	2	0.13	11	4.2	/	/	/	/	/	/	/	/	/	/
E1-167	3-23	3-3	4	α1	EAGILRFLDPRETTYPIDY	19	2	4	-0.49	23	6.6	λ	5-37	3	MISPTRASKV	10	2	0	0.01	26	8.2	X	X	/	/	/	/	X	X	/	X
E1-169	3-23	6-19	4	γ2	ESGSVAFYPFDN	12	0	2	-0.32	21	6.4	κ	1-5	4	QQYSSYFT	8	0	0	-1.14	15	5.8	/	/	/	/	/	/	/	/	/	/
E1-170	1-18	1-26	3	α1	ATFSGNYKGGGDAFDI	16	1	2	-0.32	21	6.1	λ	2-23	3	CSYAGSNTLI	10	0	0	0.51	9	3.0	/	/	/	/	/	/	/	/	/	/
E1-171	3-7	2-2	4	α1	GYCSSRSCYMIGVHYFDS	18	2	1	-0.04	21	6.4	λ	2-14	3	SSYTRSPRGLV	11	2	0	-0.67	19	6.3	/	/	/	/	/	/	/	/	/	/
E1-173	1-18	3-3	4	α1	VILQSYDFWNASPSGVDY	18	0	2	-0.03	20	5.8	λ	1-47	3	AAWDDSLSGPV	11	0	2	0.01	14	4.7	X	X	/	/	/	/	/	/	/	/
E1-174	4-59	6-13	4	α1	HADHSSWSYLDY	12	2	2	-1.14	14	4.3	κ	3-20	1	QQYGSSPRT	9	1	0	-1.90	5	1.9	/	/	/	/	/	/	/	/	/	/
E1-175	1-69/1-69D		2	α1	KKGAIFWYFDL	11	2	1	0.16	11	3.4	λ	3-21	3	QVWDTSSDHMV	11	1	2	-0.60	7	2.4	X	X	/	/	/	/	/	X	/	X
E1-177	3-48	3-10	5	γ2	VGGLGQFRF	9	1	0	0.49	34	10.8	κ	1-5	1	QQYNRYSRT	9	2	0	-2.62	12	4.6	/	/	X	X	X	X	X	X	X	X
E1-178	3-64D	3-22	6	α1	GHWETGTNYYFHGMDV	16	2	2	-0.88	24	7.1	κ	2-30	5	MQATHWPIT	9	1	0	-0.27	6	2.2	/	/	/	/	/	/	/	/	/	/
E1-181	1-46		5	α2	GRGLDP	6	1	1	-1.10	8	2.6	κ	2-30	1	MQSTQWPAT	9	0	0	-0.89	5	1.8	/	/	/	/	/	/	/	/	/	/
E1-185	4-59	1-26	4	α1	HASGTYFRAIDY	12	2	1	-0.40	16	5.1	κ	3-20	1	QQFGGAPWT	9	0	0	-0.71	9	3.0	X	X	/	/	/	/	/	/	/	/
E1-191	1-69/1-69D	3-10	6	γ1	PSQPPLFHGDYYYCLDV	17	1	2	-0.38	17	5.0	κ	1-5	4	QQYDHYAFT	9	1	1	-1.38	25	8.4	/	/	/	/	/	/	/	/	/	/
E1-195	4-4	6-19	4	α2	SSGSYVIGH	9	1	0	0.11	19	5.9	κ	3-20	2	QQYDHSLMYT	10	1	1	-1.21	12	4.3	/	/	/	/	/	/	/	/	/	/
E2-102	3-72	3-3/3-22	4	α2	SSPGSAVVTDFDY	13	0	2	-0.03	13	3.8	λ	1-44	3	AAWDDSLNGMV	11	0	2	0.08	21	7.3	/	/	/	/	/	/	/	/	/	/
E2-103	4-59	5-24/2-21	5	α1	RQQFINGGYFDP	12	1	1	-1.01	36	11.1	λ	3-1	1	QTWDSNTAI	9	0	1	-0.81	27	9.4	/	/	/	/	/	/	/	/	/	/
E2-105	1-69/1-69D	5-5/5-18	5	α1	GNRGLPLEN	9	1	1	-1.09	14	4.4	κ	3-11	1	QQRGNWPPWT	10	1	0	-2.11	4	1.5	X	X	/	/	/	X	X	X	X	X
E2-107	3-23	3-16/3-9	5	γ2	DQNVAMPAGEFDV	13	0	3	-0.16	15	4.5	λ	2-8	3	SSYAGTNIGV	10	0	0	0.26	13	4.5	/	/	/	/	/	/	/	/	/	/
E2-109	4-61	4-11/4-4	5	α1	HYSNYDRNWFDP	12	2	2	-2.07	12	3.6	κ	1D-39/1-39	3	QQSYSVPFT	9	0	0	-0.58	9	3.4	/	/	/	/	/	/	/	/	/	/
E2-110	3-23	5-12	3	α1	DRLSGNTTPDNFDV	14	1	3	-1.10	20	6.0	κ	1-33/1D-33	2	QQYNDLPRYT	10	1	1	-1.96	10	3.7	/	/	/	/	/	/	/	/	/	/
E2-111	4-30-4	6-19/5-12	5	α1	EVAINNWFDP	10	0	2	-0.32	7	2.2	λ	3-21	3	QVWDTRINPVI	11	1	1	-0.07	23	8.0	/	/	/	/	/	/	/	/	/	/
E2-113	4-31	2-2/2-15	6	α1	GEGYCSRSNCHPGNSGLDV	19	2	2	-0.82	19	5.4	κ	3-20	1	QQFADSPWT	9	0	1	-1.10	9	3.0	/	/	/	/	/	/	/	/	/	/
E2-117	1-18	2-2	6	α1	DDRGYCSSPTCSLYYYYYGMDV	22	1	3	-0.61	17	4.8	κ	3-11	4	QQRYNRLT	8	2	0	-2.21	9	3.4	/	/	/	/	/	/	/	/	/	/
E2-119	4-59	2-2	6	α2	EPPLYCSTTSCHYGMDV	17	1	2	-0.26	7	2.0	κ	1D-39/1-39	1	QQSYSTPWT	9	0	0	-1.53	16	6.1	/	/	/	/	/	/	/	/	/	/
E2-121	4-39	3-9	5	α1	LRRYYDSRADF	11	3	2	-1.41	18	5.5	λ	2-8	3	SSYASSNNYVV	11	0	0	-0.24	16	5.5	/	/	X	/	/	/	/	/	/	/
E2-122	3-7	2-21	4	α2	DPHYCGDTGCQKD	13	2	3	-1.58	12	3.6	κ	1-5	2	QQYHSNPPYT	10	1	0	-2.10	17	6.1	/	/	/	/	/	/	/	/	/	/
E2-123	3-7	1-26	3	α2	GRGSLGYL	8	1	0	-0.03	13	4.1	κ	2-30	1	MQGTHWPWT	9	1	0	-1.11	5	1.8	/	/	/	/	/	/	/	/	/	/
E2-124	3-23	3-10/2-21	6	γ1	SFDWRYYSMDV	11	1	2	-0.70	16	4.9	κ	1D-39/1-39	1	QQTYTTPWT	9	0	0	-1.51	10	3.0	/	X	/	/	/	/	/	/	/	/
E2-126	3-9	3-22	4	γ1	DRGGYYLILDH	11	2	2	-0.55	12	3.7	κ	1D-39/1-39	3	QQSYSTPFT	9	0	0	-1.12	7	2.7	/	/	/	/	/	/	/	/	/	/
E2-128	5-51	4-11/4-4	4	γ2	HHDYSHYFFDY	11	3	2	-1.43	26	8.0	κ	1-17	2	LQYHIYPFT	9	1	0	-0.06	13	4.8	/	/	/	/	/	/	/	/	/	/
E2-129	3-30	2-15/4-23/6-19	6	α1	VRSTSGWYYSYYAMDV	16	1	1	-0.34	15	4.4	λ	2-14	3	SSYTSINTRV	10	1	0	-0.44	19	7.3	/	/	/	/	/	/	/	/	/	/
E2-131	1-18	3-3	4	α2	GSGSSHPGLDY	11	1	1	-0.85	22	6.8	λ	1-40	3	QSYDSSLSVVV	11	0	1	-0.32	29	10.0	/	/	/	/	/	/	/	/	/	/
E2-135	3-48	4-11/4-4	6	γ2	SGTTVATYYFYAMDV	15	0	1	0.40	29	8.6	κ	3-20	4	QQYGRSFLT	9	1	0	-0.90	20	6.7	/	/	/	/	/	/	/	/	/	/
E2-136	4-30-4/4-31	6-19/1-26/2-8	2	γ1	DFRPRQ	6	2	1	-2.47	50	16.7	λ	2-8	1	NSYASGNNLL	10	0	0	-0.44	29	10.1	X	X	/	/	/	X	X	X	X	X
E2-137	3-15	4-17	4	α2	MTTVTTVDH	9	1	1	0.09	25	7.7	κ	2-30	1	MQGTHWPPT	9	1	0	-1.19	4	1.4	/	/	/	/	/	/	/	/	/	/
E2-140	3-33	1-26/2-21	4	α1	GREMTALVGGFDY	13	1	2	-0.02	10	3.0	κ	1D-39/1-39	2	QQTYIPPYT	9	0	0	-1.08	8	3.1	/	/	/	/	/	/	/	/	/	/
E2-141	4-61	3-22	4	α1	DRFYDSNGYYLPD	13	1	3	-1.43	15	4.4	λ	1-44	1	AVWDASLNGYV	11	0	1	0.49	21	7.3	X	X	/	/	/	X	X	X	X	X
E2-142	3-30	6-19/6-25	6	α2	GRVQTSGGGRYSGMDV	16	2	1	-0.71	15	4.4	κ	2D-28/2-28	4	MQPLQTPHT	9	1	0	-1.01	9	3.3	/	/	/	/	/	/	/	/	/	/
E2-145	3-23	3-3	6	α1	EDSEWLLYGPQLYNGMDV	18	0	4	-0.57	12	3.4	κ	2D-28/2-28	2	MQALQTPRT	9	1	0	-0.78	6	2.2	/	/	/	/	/	/	/	/	/	/
E2-147	3-9	6-19	5	α1	DGAVVGGFDFPGSGVGALAS	20	0	2	0.74	15	4.5	κ	3-20	3	QQYGISPFS	9	0	0	-0.51	17	6.3	/	X	X	/	/	/	/	/	/	/
E2-148	3-30	1-26/3-10/5-24	6	α2	ITGGASYYNGLDV	13	0	1	0.15	9	2.7	κ	1D-39/1-39	4	QQSSSTPLT	9	0	0	-0.96	11	4.2	/	/	/	/	/	/	/	/	/	/
E2-154	3-30	6-13	5	γ2	GGSSTFGFDP	10	0	1	-0.30	11	3.4	λ	3-21	3	QVWDTSIDHVV	11	1	2	0.09	27	9.4	/	/	/	/	/	/	/	/	/	/
E2-155	3-48	4-17	4	α2	RDGDYPY	7	1	2	-2.30	8	2.5	κ	4-1	4	QQYYSTPLT	9	0	0	-1.07	4	1.4	/	/	/	/	/	/	/	/	/	/
E2-159	4-31	6-19	4	α1	GGGVSVAGIDF	11	0	1	1.05	13	4.0	κ	3-11	3	QYRSTWPPRVT	11	2	0	-1.45	8	2.9	X	/	/	/	X	X	X	X	X	X
E2-163	4-4	2-15	4	α1	VTDGSGGPSYYFDY	14	0	2	-0.64	19	5.7	κ	3-11	3	QHRILRPPGFT	11	3	0	-0.81	11	4.1	/	/	/	/	/	/	/	/	/	/
E2-166	3-13	1-1/1-20	6	α1	GLGPFDERQTFGMDV	15	1	3	-0.43	14	4.2	κ	1-27	3	QNYNSAPPKAT	11	1	0	-1.53	8	3.0	/	/	/	/	/	/	/	/	/	/
E2-167	3-23	3-16/3-9	4	α1	DRDYYESTYYFDN	13	1	4	-1.99	17	5.2	κ	4-1	4	QQYFYTPLT	9	0	0	-0.67	10	3.5	/	/	/	/	/	/	/	/	/	/
E2-173	3-7	2-8/3-16	4	γ1	LGPGVKSDY	9	1	1	-0.43	15	4.7	κ	1-5	1	QQYNSYSQT	9	0	0	-2.10	11	3.8	X	/	/	X	X	X	X	X	X	X
E2-176	4-59	3-22	4	γ1	STKYFYDRSGYYPTKPFDY	19	3	2	-1.41	13	3.9	κ	3-20	1	QQYGSSPWT	9	0	0	-1.50	7	2.3	X	/	X	X	X	X	X	X	X	X
E2-178	1-18	4-17	5	α2	SGGGPLPNHYGDGNWFDP	18	1	2	-1.13	23	6.6	λ	2-23	3	CSYGGGGSLWV	11	0	0	0.46	17	6.4	/	/	/	/	/	X	X	X	/	X
E2-183	4-34	4-23/3-9/1-7	5	α2	RTRFLPVDK	9	3	1	-0.88	20	6.3	κ	3-20	1	QHYGSWT	7	1	0	-1.54	13	5.0	X	X	/	/	/	/	/	X	/	/
E2-186	3-74	5-18/4-11/4-4	6	γ1	APYSRKNNYYYFAMDV	16	2	1	-0.88	19	5.6	κ	1D-39/1-39	5	QQSFSTPIT	9	0	0	-0.48	10	3.8	/	/	/	/	/	/	/	/	/	/
E2-187	4-61	1-14/4-11/4-4	6	γ1	FPPDNLYRHSLVDV	14	2	2	-0.34	29	9.0	κ	3-20	3	QQYSISPRT	9	1	0	-1.36	22	8.0	/	/	/	/	/	X	X	X	/	/
E2-189	4-39	6-13	4	α1	GLGMAALGHFDY	12	1	1	0.56	20	6.0	κ	4-1	2	HQYFTAPDT	9	1	1	-1.10	11	3.9	X	/	/	/	/	/	/	X	/	X
E2-192	3-23	6-19	6	α2	AHSSGWYPFYYYGLDV	16	1	1	-0.26	9	2.6	λ	1-40	3	QSYDSSLRTSI	11	1	1	-0.76	23	7.9	/	/	/	/	/	X	/	/	/	/
E2-195	1-46	6-6/6-13/5-24	4	α2	GRIAHADS	8	2	1	-0.54	27	8.6	κ	1-9	4	QQLDYYRLA	9	1	1	-0.91	21	7.3	/	/	/	/	/	X	X	X	X	X
E3-110	3-23	6-19/3-10	4	α1	EAGFALPFDF	10	0	2	0.68	23	7.1	κ	3-11	1	QQRRTWWT	8	2	0	-2.40	11	4.3	/	/	/	/	/	/	/	/	/	/
E3-111	3-30	2-21	6	α1	ARSHCGGDCYSTYGMDV	17	2	2	-0.46	5	1.5	κ	3-11	4	QQRSNWPLT	9	1	0	-1.69	2	0.7	/	/	/	/	/	/	/	/	/	/
E3-111	4-59	4-17	6	γ2	YSYGDHGYYYYILDV	15	1	2	-0.47	26	7.7	κ	3-11	4	QQRSNWPLT	9	1	0	-1.69	2	0.7	/	/	/	/	/	/	/	X	X	/
E3-113	5-51	4-17	4	α1	QSYGDSRVDY	10	1	2	-1.54	1	0.3	λ	2-14	3	SSYTSSSTLHVV	12	1	0	0.19	5	1.6	/	/	/	/	/	/	/	/	/	/
E3-118	3-7	7-27	5	α2	ITGDKSFVF	9	1	1	0.56	10	3.2	κ	1-16	3	QQYKSYPLT	9	1	0	-1.42	9	3.4	/	/	/	/	/	/	/	/	/	/
E3-120	3-43	2-8/7-27	5	α1	GLYSTNWWRALGS	13	1	0	-0.37	21	6.4	κ	2D-28/2-28	5	MQALHTPHIT	10	2	0	-0.09	9	3.2	/	/	/	/	/	/	/	/	/	/
E3-124	4-31	3-22	4	α1	DRSGIGSAYYSYFES	15	1	2	-0.69	20	5.8	κ	3-15	1	QQYNSWPRT	9	1	0	-2.26	10	3.8	/	/	/	/	/	/	/	/	/	/
E3-130	5-51	5-12	4	γ2	RGYSAYDGQFDY	12	1	2	-1.33	13	4.0	λ	1-51	3	GTWDSSVSVWL	11	0	1	0.31	7	2.3	/	/	/	/	/	/	/	/	/	/
E3-139	3-74	1-26	5	α2	DLTWELSS	8	0	2	-0.33	22	7.3	λ	1-51	3	GAWDSGLSAAL	11	0	1	0.56	9	3.0	/	/	/	/	/	/	/	/	/	/
E3-144	3-66	3-16	4	α1	FPAWGSYQTSCDY	13	0	0	1.00	8	2.7	κ	1D-39/1-39	5	QQSYSIPIT	9	0	0	-0.36	12	4.4	/	/	/	/	/	/	/	/	/	/
E3-146	3-23	1-26	4	α2	DKGSRGLGATPDY	13	2	2	-1.18	11	3.3	λ	2-11	3	CSYAGSYTLV	10	0	0	0.70	13	4.4	/	/	/	/	/	/	/	/	/	/
E3-147	3-30	1-26	4	α2	KFGGSGSYYVAFHY	14	2	0	-0.16	5	1.5	λ	1-40	3	HSYDSSLSGWV	11	1	1	-0.41	7	2.3	/	/	/	/	/	/	/	/	/	/
E3-148	3-30	3-16	4	α1	PLGGGPPRPFDY	12	1	1	-0.86	28	8.6	κ	3-15	4	QQYNNWPLT	9	0	0	-1.63	10	3.8	/	/	/	/	/	/	/	/	/	/
E3-154	3-21	4-17	4	γ1	DRVAAKHGDSFDY	13	3	3	-1.08	16	4.8	κ	3D-20	5	QQYGSSPIT	9	0	0	-0.90	11	4.2	/	/	/	/	/	/	/	/	/	/
E3-156	3-74	3-22	4	γ1	EGYSMIVVPQLRADY	15	1	2	0.00	4	1.2	κ	1-33/1D-33	2	QQYDTLPYT	9	0	1	-1.37	6	1.9	/	/	/	/	/	/	/	/	/	/
E3-157	3-7	5-24	6	α1	DSPDNGYHFGYYGLDV	16	1	3	-0.87	20	5.9	κ	2D-28/2-28	5	MQALQTPNT	9	0	0	-0.67	12	4.3	/	/	/	/	/	/	X	X	X	X
E3-159	3-49	3-3	4	α1	YYDSWNGYPRGDY	13	1	2	-1.87	11	3.3	κ	1D-39/1-39	3	QQSYVIQLT	9	0	0	-0.09	12	4.6	/	/	/	/	/	/	/	/	/	/
E3-165	3-23	4-17	3	α1	DPTGDYVGAFDM	12	0	3	-0.35	27	8.3	κ	3-20	3	QQYGSSFT	8	0	0	-1.03	13	4.9	/	/	/	/	/	/	/	/	/	/
E3-166	3-48	5-5/5-18	4	α1	GEVSGYGYGFFDY	13	0	2	-0.27	10	3.0	κ	3-15	5	QQYNNWPPVT	10	0	0	-1.59	4	1.5	/	/	/	/	/	/	/	/	/	/
E3-167	3-30	6-19	6	α1	ETQWLANYYSYGMDV	15	0	2	-0.60	3	0.9	λ	2-23	1	CSYAGSSNV	9	0	0	0.10	4	1.3	/	/	/	/	/	/	/	/	/	/
E3-168	3-30	2-8	6	α1	DLDIALVEAALDV	13	0	4	1.21	24	7.3	κ	3-20	5	QQYSSSPPIT	10	0	0	-1.01	22	7.9	X	X	/	/	/	/	/	/	/	/
E3-170	3-74	2-21	3	α2	DYPGEHNAFDI	11	1	3	-1.04	14	4.3	κ	3-20	2	QQYDTSPRVYT	11	1	1	-1.56	8	2.9	/	/	/	/	/	/	/	/	/	/
E3-179	4-59	1-26	4	α2	HASGSYYGRFEY	12	2	1	-1.08	14	4.3	κ	3-15	1	LQYNNWPPWT	10	0	0	-1.37	6	2.3	X	/	X	/	/	/	/	/	/	/
E3-183	4-4	5-5/5-18	4	γ2	GEYSYGYFDY	10	0	2	-1.10	15	4.9	κ	1-5	4	QQFNSYSPLT	10	0	0	-0.91	7	2.7	/	/	/	/	/	/	/	/	/	/
E3-189	1-46	3-16	4	α2	LAVWGGGY	8	0	0	0.80	22	6.9	κ	1-17	4	LQHNDYPFT	9	1	1	-1.19	10	3.8	/	/	/	/	/	/	/	/	/	/
E3-191	3-64	5-24	6	α2	DGQRWLQNYYYGMHV	15	2	1	-1.16	21	6.3	λ	1-40	1	QSYDSSLSGYV	11	0	1	-0.47	14	4.6	/	/	/	/	/	/	/	/	/	/
L1-106	3-30	2-8	3	α1	TNTILMLYAMRGVFDI	16	1	1	0.91	13	3.8	λ	2-14	3	SSYRTSSTLNWV	12	1	0	-0.57	4	1.3	X	X	X	/	/	/	/	/	/	X
L1-107	3-72	4-11/4-4	6	α2	GYSNYVGVHYGLDV	14	1	1	0.02	10	3.0	λ	2-8	3	YSYAGINNGWV	11	0	0	-0.15	5	1.7	/	/	/	/	/	/	/	/	/	/
L1-108	4-39	6-19	4	α1	RPHNYSSRWAFDY	13	3	1	-1.64	12	3.6	λ	2-14	1	SSYTSSSTPSV	11	0	0	-0.45	7	2.3	/	/	/	/	/	X	X	X	X	X
L1-109	1-18	1-26	5	γ2	VVGIVGPKGWFDP	13	1	1	0.55	13	3.9	κ	3-20	5	QQYGDSPIT	9	0	1	-1.20	8	3.0	/	/	/	/	/	/	X	X	/	X
L1-111	3-30	6-19	4	γ1	DPWRYSTGWYGIDY	14	1	2	-1.19	24	7.2	λ	7-46	3	LLSYSGDWV	9	0	1	0.46	19	6.4	X	/	/	/	/	X	X	X	X	X
L1-113	4-59	6-13	5	γ1	SPWRQQLHF	9	2	0	-1.27	16	5.3	κ	1D-39/1-39	3	QQGYSTPFT	9	0	0	-1.08	10	3.8	/	/	/	/	/	/	/	/	/	/
L1-114	3-23	3-9	4	γ1	DDWDYFHY	8	1	3	-1.80	27	8.6	κ	3-11	5	QQRANWPPIT	10	1	0	-1.35	15	5.7	/	/	/	/	/	X	/	X	X	X
L1-117	3-7	5-5/5-18/3-10	4	α2	GPSYGLRTDFFDS	13	1	2	-0.62	38	11.4	κ	2D-28/2-28	4	MQSVQDPFT	9	0	1	-0.52	33	11.8	/	/	/	/	/	/	/	X	/	X
L1-118	3-23	6-13	3	γ1	IQQLILDAFDI	11	0	2	1.06	10	3.1	λ	3-21	3	QVWDSSSDHPDVV	13	1	3	-0.73	9	2.0	/	/	/	/	/	X	X	X	X	X
L1-120	3-30	5-24	6	α2	DGGAYQVFNYKYYGMDV	17	1	2	-0.55	14	4.3	λ	3-21	3	QVWDTSSDQMA	11	0	2	-0.85	17	5.8	/	/	/	/	/	X	X	X	X	X
L1-121	3-48	3-10	4	α1	GLKISASGSRIGVADN	16	2	1	0.10	20	5.9	κ	4-1	2	QQYFSTPVT	9	0	0	-0.57	11	3.9	X	X	X	X	X	X	X	X	X	X
L1-133	3-53	3-22/2-21/3-3	6	α1	ENYSYGLDV	9	0	2	-0.70	16	5.1	λ	1-44	3	AAWDDSLNGWV	11	0	2	-0.17	8	2.7	/	/	/	/	/	/	/	X	/	/
L1-139	4-31	1-20/1-7	4	γ1	GRGYNWNPFDY	11	1	1	-1.65	5	1.5	λ	1-40	1	QSYDSSLSGPV	11	0	1	-0.50	2	0.7	X	/	X	X	X	X	X	X	X	X
L1-142	4-38-2	2-15	4	γ1	EYCSGGSCPIDY	12	0	2	-0.34	3	0.9	λ	1-47	3	AAWDDSLSGWV	11	0	2	0.07	7	2.3	/	/	/	/	/	/	/	X	/	X
L1-143	4-34	6-25	6	α1	VYRVITGYLYYGMDV	15	1	1	0.54	20	6.0	κ	2D-28/2-28	2	MQALQTPYT	9	0	0	-0.42	12	4.2	X	X	/	X	X	X	X	X	X	X
L1-145	3-48	3-22/2-21	6	α2	LLGGDYHYYHHMDA	14	3	2	-0.71	21	6.4	κ	1D-39/1-39	1	QQTYHTPWT	9	1	0	-1.79	25	9.6	/	/	/	/	/	/	/	/	/	/
L1-148	4-59	4-17	6	γ1	HIQTRYGDYMTDYYFYAMDV	20	2	3	-0.65	16	4.6	κ	1D-39/1-39	2	QQTYTTPYT	9	0	0	-1.56	17	6.5	/	/	/	/	/	/	/	/	/	/
L1-152	3-30	6-13	4	α2	SMWGVPAAGTHY	12	1	0	0.03	27	8.1	λ	1-51	3	GAWDTTLNLVL	11	0	1	0.70	15	5.0	/	/	/	/	/	/	/	/	/	/
L1-153	4-31	6-19	4	α1	VVSGSSSPYFDY	12	0	1	-0.19	23	7.2	κ	1D-39/1-39	5	QQGYQTPVT	9	0	0	-1.22	43	16.5	/	/	/	/	/	/	X	X	X	X
L1-154	3-48	3-16	4	γ1	DVTPFVWGSYQDH	13	1	2	-0.63	31	9.4	κ	3-15	4	QQYDNWPPVT	10	0	1	-1.59	13	4.9	X	/	/	/	/	/	/	X	/	X
L1-156	3-64D	1-26	5	γ1	GMEWELLGPFEC	12	0	3	0.08	13	4.0	κ	2D-28/2-28	1	MHALQSPT	8	1	0	-0.29	6	2.1	X	/	/	X	/	X	X	X	X	X
L1-158	4-31	2-8/3-10	6	γ1	GMKALNQLLFEWHFYAMDV	19	2	2	0.26	52	14.6	λ	3-21	3	QVWDSSSDHWV	11	1	2	-0.86	6	2.0	/	/	/	/	/	/	/	/	/	/
L1-161	3-33	3-10	4	γ3	DGLNYFGSGDWYKADTLDY	19	1	4	-0.88	19	5.5	κ	1-27	4	QKYNSAPLT	9	1	0	-1.08	13	4.6	X	X	/	/	/	/	/	X	X	X
L1-162	3-30-3	4-23/4-17	6	γ1	SQKPYGAHYYYGMDV	15	2	1	-0.97	3	0.9	λ	3-21	3	QVWDSSSDHWV	11	1	2	-0.86	0	0.0	/	/	X	X	X	X	X	X	X	X
L1-164	3-74	3-10	4	γ1	GWFGTVGY	8	0	0	0.36	13	4.1	λ	2-14	3	SSYTDDSTVI	10	0	2	-0.34	14	4.7	X	X	/	X	X	X	X	X	X	X

L1-165	1-2	3-9	4	γ1	SNILTGYYYFDY	12	0	1	-0.25	3	0.9	λ	3-1	3	QAWDSSTAL	9	0	1	-0.31	3	1.0	X	/	/	/	/	X	X	X	X	X
L1-166	3-21	2-2	5	α1	SSRWSGAIDP	10	1	1	-0.70	12	3.7	λ	1-51	3	GTWDSSLSDWV	11	0	2	-0.39	10	3.3	X	/	/	X	X	X	X	X	X	X
L1-168	4-39	6-19	6	α1	IVAGYFRYNDMDV	13	1	2	0.11	26	7.7	κ	3-11	4	HQRSNWPRT	9	3	0	-2.58	6	2.2	X	X	/	X	X	X	X	X	X	X
L1-170	5-51	2-15	4	α1	QEIGCPGGSCYPDS	14	0	2	-0.59	16	4.8	λ	1-44	3	AAWDDALKAWV	11	1	2	0.23	23	7.7	/	/	/	/	/	/	/	/	/	/
L1-175	3-23	3-16	4	α1	VIGDTIMLTADY	12	0	2	0.88	23	7.1	κ	2D-28/2-28	3	MQALQSPT	8	0	0	-0.33	22	5.3	/	/	/	/	/	/	/	/	/	/
L1-181	3-23	3-16	5	γ1	GRGGERA	7	2	1	-1.70	21	6.7	κ	1-5	1	QQYTSYLWT	9	0	0	-0.99	19	7.3	/	/	/	X	X	X	X	X	X	X
L1-186	3-23	2-8/5-12/4-23	3	γ1	GYGTKSQASNI	11	1	0	-0.82	13	4.0	λ	3-21	3	QVWDSSSDHVV	11	1	2	-0.40	7	2.4	/	/	/	/	/	/	/	X	/	/
L1-187	1-18	2-15	6	α1	DLYCSGGSCWGDAMDV	16	0	3	0.08	19	5.6	κ	3-11	2	QQRSNWPPYT	10	1	0	-2.19	12	4.5	X	/	/	/	/	X	X	X	X	X
L1-188	3-21	6-19	6	α2	DAVAGYYYYYGMDV	14	0	2	-0.03	13	3.9	κ	3-11	3	QQRSNWPPFT	10	1	0	-1.78	17	6.7	X	/	/	/	/	X	X	X	X	X
L1-190	3-23	6-6	6	γ1	DAQSSADSWYRSQPDYYYYGMDV	23	1	4	-1.23	31	8.5	κ	1D-39/1-39	3	QQSYSSPFT	9	0	0	-1.13	19	7.2	/	/	/	/	/	/	/	X	/	X
L1-194	3-23	1-26	4	α1	AGSHSGSYRPFDY	13	2	1	-1.08	21	6.4	λ	3-21	1	QVWDSSTEHYV	11	1	2	-0.89	13	4.4	/	/	/	/	/	/	/	/	/	/
L2-108	3-15	3-22	4	α1	VYYDTSPGTLRSYFEY	16	1	2	-0.68	21	6.1	λ	1-47	3	AAWDYSLSGRV	11	1	1	-0.05	17	5.7	/	X	/	/	/	/	/	/	/	/
L2-109	4-4	2-15/3-22	4	γ2	AWRISGSVFDY	11	1	1	0.10	18	5.5	κ	1D-39/1-39	1	QQNYSPPWT	9	0	0	-1.93	6	2.3	/	/	X	/	X	/	/	X	X	X
L2-112	3-21	6-19/6-13/5-24	4	γ1	DRDSTSIGFDY	11	1	3	-1.06	37	11.4	λ	3-21	3	QVWDSRSDHVV	11	2	2	-0.74	26	8.8	X	/	X	X	X	X	X	X	X	X
L2-117	3-49	3-10	4	γ1	DKERRGYYFDY	11	3	3	-2.26	12	3.7	λ	1-44	3	ATWDDSLTGRL	11	1	2	-0.51	9	3.0	/	/	/	/	/	/	/	/	/	/
L2-122	4-59	3-10	6	α1	GIPDFGSQTYRGADV	15	1	2	-0.49	17	5.1	κ	1D-39/1-39	1	QESYSLSWT	9	0	1	-0.94	34	6.7	/	/	/	/	/	/	/	/	/	/
L2-124	3-9	1-1/1-26/1-20	4	γ1	EGGYWTYYFDF	11	0	2	-0.70	14	4.3	λ	2-23	3	SSYAGSRTSM	10	1	0	-0.64	12	4.0	/	/	/	/	/	/	/	X	X	X
L2-125	3-30	3-22	6	γ1	EGFEANSNSPSVGPLGLDV	19	0	3	-0.19	14	4.0	κ	1-27	3	QNYNSAPFT	9	0	0	-1.14	6	2.3	/	/	/	/	/	/	/	/	/	/
L2-126	3-7	3-3/3-22	3	α1	DQPNPSGYPTDPFDI	15	0	3	-1.32	28	8.3	κ	3-15	2	QQYNSWPPYT	10	0	0	-1.87	16	6.1	/	/	/	/	/	/	/	/	/	/
L2-129	4-31	3-10/3-22	4	α1	RPRITARSLSY	11	3	0	-0.78	46	13.9	κ	1-5	4	QQYSDYSPLT	10	0	1	-1.32	40	15.2	X	X	/	/	/	/	/	/	/	/
L2-130	3-72	3-10/3-22	6	α1	AHSEYHLLYGRDYYYGMDV	19	3	3	-0.74	18	5.0	λ	1-40	1	QSYDNNLNGFYV	12	0	1	-0.88	15	4.9	X	X	/	/	/	/	/	/	/	/
L2-134	1-18	6-19	6	α2	PLTTSQWPHYGMDV	14	1	1	-0.59	13	3.9	κ	2D-28/2-28	3	MQALQNVFT	9	0	0	0.37	1	0.4	X	X	/	X	X	X	X	X	X	X
L2-138	3-48	2-21/3-9	4	α1	DRDWSFDS	8	1	3	-1.84	24	7.7	λ	2-23	3	CSYGGTGISWV	11	0	0	0.50	17	5.6	X	X	/	X	X	X	X	X	X	X
L2-139	3-73	6-19	5	α1	SDGYSSGWYSS	11	0	1	-1.07	20	6.1	λ	6-57	3	QSYDINGRVL	10	1	1	-0.50	21	5.1	/	X	/	X	/	X	X	X	X	X
L2-142	3-7	1-7	5	γ1	GNDWAGTTPY	10	0	1	-1.12	29	9.0	κ	2D-28/2-28	1	MQALRGLPT	9	1	0	0.07	16	5.7	/	/	/	/	/	/	/	X	/	/
L2-144	5-51	3-22	4	γ1	SPVPGRYHFDYRGYYYFDL	19	3	2	-0.89	30	8.6	κ	3-20	4	QQYTTSHT	8	1	0	-1.80	15	5.4	X	X	X	/	/	/	/	/	/	/
L2-146	3-21	2-2/2-15	5	α1	GNGEYCNSVSCFPGWIDP	18	0	2	-0.32	25	7.3	λ	1-51	3	GTWDNSLSAWV	11	0	1	-0.15	16	5.3	/	/	/	/	/	/	/	/	/	/
L2-155	5-51	3-10	3	γ1	RGEDYSASGSGDFDI	15	1	4	-0.95	15	4.5	κ	2-30	1	MQGTHWPWT	9	1	0	-1.11	5	1.8	/	/	/	/	/	/	/	/	/	/
L2-156	1-18	3-22/3-16	4	α1	VNYYASNDYYYVFNY	15	0	1	-0.64	26	7.7	λ	2-14	1	SSYTSSSTV	9	0	0	-0.28	17	5.7	/	X	/	/	X	/	/	/	/	/
L2-160	5-51	1-7	3	α1	NNAITGTIRDAFDI	14	1	2	-0.03	18	5.4	λ	2-14	3	SSYTTSSTAV	10	0	0	-0.06	13	4.3	/	/	/	/	/	/	/	/	/	/
L2-161	4-59	3-10/3-16	3	α1	HTDYYVGGTYPHAFDL	16	2	2	-0.53	28	8.3	κ	4-1	1	QQYYNLPWT	9	0	0	-1.39	21	7.5	/	/	/	/	/	/	/	/	/	/
L2-166	4-59	3-10	3	γ1	DFRKFGELLSFSGLDI	16	2	3	0.19	25	7.4	κ	1D-39/1-39	4	QQSYSVPLT	9	0	0	-0.47	14	5.3	X	X	/	/	/	/	/	/	/	/
L2-167	4-34	3-22	4	γ1	GLRRRYYYDNTGPRVYYFDY	20	4	2	-1.43	7	2.0	κ	4-1	4	QQYYSSPLS	9	0	0	-1.09	2	0.7	X	/	X	X	X	X	X	X	X	X
L2-168	4-39	7-27	4	α2	DHTTWGRMGH	10	3	1	-1.56	12	3.7	κ	3-20	1	QHYGSSSWT	9	1	0	-1.38	9	3.0	/	/	/	/	/	/	/	/	X	/
L2-169	3-9	6-19	4	α2	GGSSDWSPCY	10	0	1	-0.80	20	6.2	κ	1-8	1	HQYDTYPWS	9	1	1	-1.87	20	6.9	X	/	/	X	X	X	X	X	X	X
L2-170	3-30/3-30-3	2-2	5	γ2	GKGEHCSSPTCLLRRHSFDP	20	5	2	-0.90	16	4.6	λ	3-21	3	QVWDGGEDHVGVI	13	1	3	-0.17	16	5.1	/	/	/	/	/	/	/	/	/	/
L2-171	4-34	6-13	5	γ1	GRSNRQQLILGRSPKPNYFDP	21	4	1	-1.36	16	4.5	κ	3-15	2	QQYNDWPPYT	10	0	1	-2.14	6	2.2	X	/	/	/	/	X	X	X	X	X
L2-173	3-7	7-27	5	α1	GGTYPGL	7	0	0	-0.14	10	3.2	κ	3-15	1	QQYDNWPNT	9	0	1	-2.44	5	1.9	/	/	/	/	/	/	/	/	/	/
L2-176	3-48	3-22	4	α1	AGDYDSGGYDIPTFDY	16	0	4	-0.82	21	6.3	κ	1D-39/1-39	2	QQSYSTPLYT	10	0	0	-1.04	14	5.2	/	/	/	/	/	/	/	/	/	/
L2-177	4-4	2-2	6	γ2	EDGHCNSPSCYRYYNNGMDV	20	2	3	-1.28	17	4.8	κ	3-20	1	QQYGSLPWT	9	0	0	-0.99	17	6.1	/	/	/	/	/	/	/	/	/	/
L2-179	3-48	4-17	4	α1	QTTVTSTIDY	10	0	1	-0.32	26	8.1	λ	1-47	3	SAWDDRLVGWV	11	1	2	-0.05	19	6.3	/	/	/	/	/	/	/	/	/	/
L2-180	3-74	6-25	5	α1	AGSAAVRRDNKNWFDP	16	3	2	-1.14	49	14.5	κ	3-11	5	QQRTRWPLT	9	2	0	-1.79	26	10.0	/	/	/	/	/	/	/	/	/	/
L2-181	4-39	2-8/3-9	5	α1	TPGGLVNWFDP	11	0	1	-0.16	30	9.2	κ	1-5	3	QQYVSYPST	9	0	0	-1.03	13	5.0	/	/	/	/	/	/	/	/	/	/
L2-186	1-18	6-19	1	γ1	AGRGAVASPGNF	12	1	0	0.07	25	7.6	κ	3-15	3	QQYNNWPLT	9	0	0	-1.63	5	1.9	/	/	/	/	/	/	/	/	/	/
L2-191	3-23D/3-23	1-7	1	γ1	EPETGTPSWFDH	12	1	3	-1.47	38	12.2	κ	1D-39/1-39	1	QQSYKTPRT	9	2	0	-2.28	19	7.3	/	/	/	/	/	/	/	/	/	/
L2-192	3-7	2-2/3-9	4	α2	SPRYALDY	8	1	1	-0.93	20	6.3	κ	4-1	2	QQYYTSPPYT	10	0	0	-1.63	12	4.2	/	/	/	/	/	/	/	/	/	/
L3-101	3-53	2-2	4	γ1	GLHCDSANCYTSSYFDY	17	1	2	-0.45	10	3.0	κ	3-11	2	QQRSMWPPGYT	11	1	0	-1.54	10	3.4	X	/	/	/	/	/	/	/	/	/
L3-103	3-48	2-15	3	α2	DLRYSARLDTFDM	13	2	3	-0.63	24	7.2	κ	3-20	2	QQYDASPYT	9	0	1	-1.60	17	6.3	X	X	/	/	/	/	/	/	/	/
L3-104	3-33	2-15/2-21	4	γ1	DLKYGGFEFDY	11	1	3	-0.76	19	5.9	λ	7-43	3	VLYFGGGQGM	10	0	0	0.63	17	5.7	/	/	/	/	/	/	/	/	/	/
L3-107	4-61	1-26	6	α2	SRIRGYIVGAGHYYYGMDV	19	3	1	-0.12	3	0.9	κ	1D-39/1-39	3	QQSYSNFPFT	10	0	0	-1.01	4	1.5	X	X	/	/	/	X	X	X	X	X
L3-112	3-7	1-20/1-7	4	γ2	DRGYNWDHGPTRFDY	15	3	3	-2.00	17	5.1	λ	2-14	1	CSYTSNNSRV	10	1	0	-0.92	21	7.0	/	/	/	/	/	X	X	X	/	X
L3-114	3-30-3	3-10	4	α1	SYYHGSGSHFYLFYFDY	17	2	1	-0.44	6	1.8	κ	3-11	1	QQRWT	5	1	0	-2.62	3	1.2	X	X	X	X	X	X	X	X	X	X
L3-115	3-15	1-26/3-16	4	γ1	GELLDY	6	0	2	-0.18	19	6.0	κ	1D-39/1-39	2	QQSYSIPFT	9	0	0	-0.54	14	5.4	/	/	/	/	/	/	/	X	/	/
L3-116	3-15	3-22	3	γ1	EECYYDSRGYCYHYDAFHI	19	3	4	-0.97	7	2.1	κ	1D-39/1-39	4	HQTYGLPLT	9	1	0	-0.42	14	5.4	/	/	/	/	/	/	/	/	/	/
L3-121	3-15	4-23/4-17	4	γ1	DGDYGVPYCFDY	12	0	3	-0.61	1	0.3	λ	1-51	3	GAWDSGLSAGGV	12	0	1	0.33	16	5.3	/	/	/	/	/	/	/	/	/	/
L3-121	3-15	4-23/4-17	4	γ1	DGDYGVPYCFDY	12	0	3	-0.61	1	0.3	κ	3-20	2	QQYGSSPAYT	10	0	0	-1.21	2	0.4	/	/	/	/	/	/	/	/	/	/
L3-122	3-9	1-26	2	γ1	ESGMGGTSGWFFDL	14	0	2	-0.04	26	7.8	κ	1D-39/1-39	1	QQTYTAPWT	9	0	0	-1.23	17	6.1	/	/	/	/	/	/	/	/	/	/
L3-124	1-18	4-17	4	γ1	GDYGDSFDY	9	0	3	-1.32	20	6.2	κ	1D-39/1-39	2	QQSYSAPGT	9	0	0	-1.20	11	3.8	/	/	/	/	/	/	/	/	/	/
L3-127	4-59	3-10	6	γ3	GGSGSYFQAQYYGLDV	16	0	1	-0.31	32	9.4	κ	3-15	2	QQFSNWPPPPRIS	13	0	0	-3.50	9	3.8	X	X	X	X	X	X	X	X	X	X
L3-129	1-18	2-2	4	γ3	GAYCSSTSCHDY	12	1	1	-0.50	2	0.6	κ	1-16	2	QQYNSYPYT	9	0	0	-1.94	1	0.4	/	/	/	/	/	/	/	/	/	/
L3-132	3-30-3	3-9	4	α1	DPAVGSPRYNILTGYIAFFDY	21	1	2	0.09	2	0.6	κ	1D-39/1-39	3	QQSYSTPFT	9	0	0	-1.12	0	0.0	/	X	/	/	/	/	/	/	/	/
L3-133	3-23		5	α2	DRDADS	6	1	3	-2.33	10	3.1	κ	4-1	2	QQYYSAPYT	9	0	0	-1.36	8	2.9	/	/	/	/	/	/	/	/	/	/
L3-134	1-3	3-10	3	γ1	SQPQWFTDGDALDI	14	0	3	-0.64	23	6.9	λ	4-69	3	QTWGTGTYVV	10	0	0	-0.02	18	5.9	/	/	/	/	/	/	/	/	/	/
L3-135	3-48	2-15	6	γ1	DPRSGGRGYYHYYGMDV	17	3	2	-1.31	12	3.5	κ	1D-39/1-39	3	QQSYISPT	8	0	0	-0.96	7	2.7	/	/	/	/	/	/	/	/	/	/
L3-136	3-11	3-9	5	γ1	THSTSTILRLVITWFDP	17	2	1	0.32	10	2.9	λ	3-21	3	QVWDGSSDHLV	11	1	2	-0.40	7	2.4	/	/	/	/	/	/	/	/	/	/
L3-144	4-39	3-10	5	α1	QLYDSGTYYNQRFDP	15	1	2	-1.52	13	3.8	κ	3-15	4	QQYHNWPLT	9	1	0	-1.60	8	3.1	/	/	/	/	/	/	/	/	/	/
L3-147	4-61	4-23/4-17	4	α1	EDYGRPDY	8	1	3	-2.45	14	4.4	κ	1D-39/1-39	1	QQTYSIPRT	9	1	0	-1.34	6	2.3	/	/	/	/	/	/	/	/	/	/
L3-149	4-61	3-16	4	α1	KVGELRYLPSFDY	13	2	2	-0.48	19	5.7	λ	1-47	3	AAWDDSLSGHVV	12	1	2	0.23	7	2.3	/	/	/	/	/	/	/	/	/	/
L3-152	3-48	2-8	6	α1	GPRVYYHGLDV	11	2	1	-0.36	17	5.2	κ	1-9	1	QQFNRDPWT	9	1	1	-2.10	9	3.4	X	/	/	/	/	/	/	/	/	/
L3-166	4-39	2-8	4	γ1	SYAYYFDY	8	0	1	-0.61	15	4.7	κ	3-15	4	QQYNNWPLLT	10	0	0	-1.09	4	1.5	/	/	/	/	/	/	/	/	/	/
L3-175	3-48		4	α1	DSLTHFDY	8	1	2	-0.80	9	2.9	κ	1D-39/1-39	1	QQSYNTPPWT	10	0	0	-1.81	5	1.5	/	/	/	/	/	/	/	/	/	/
L3-179	3-23	2-21/4-17	1	γ1	NTAVTGTEYFQH	12	1	1	-0.73	23	7.0	κ	3-11	1	QQRSHWPRT	9	3	0	-2.58	9	3.4	/	/	/	/	/	/	/	/	/	/
L3-185	4-59	3-16	6	γ1	DRRGLWGMDV	10	2	2	-0.78	35	10.9	κ	4-1	2	QQYFATPYT	9	0	0	-0.89	27	9.7	/	/	/	/	/	/	/	/	/	/
L3-190	4-59	3-10	5	α1	GTRTYSTNWFGGWFDP	16	1	1	-0.92	32	9.5	κ	1-27	3	QEYYSVPFT	9	0	1	-0.63	26	10.0	X	X	/	/	/	/	/	/	/	/

(-) and (+) indicate the numbers of negatively and positively charged amminoacids in the IgH complementary determining region (CDR3), respectively. SM, somatic mutations in the VH and VL Ig genes. PolyR, polyreactivity. X, reactive; /, non reactive.

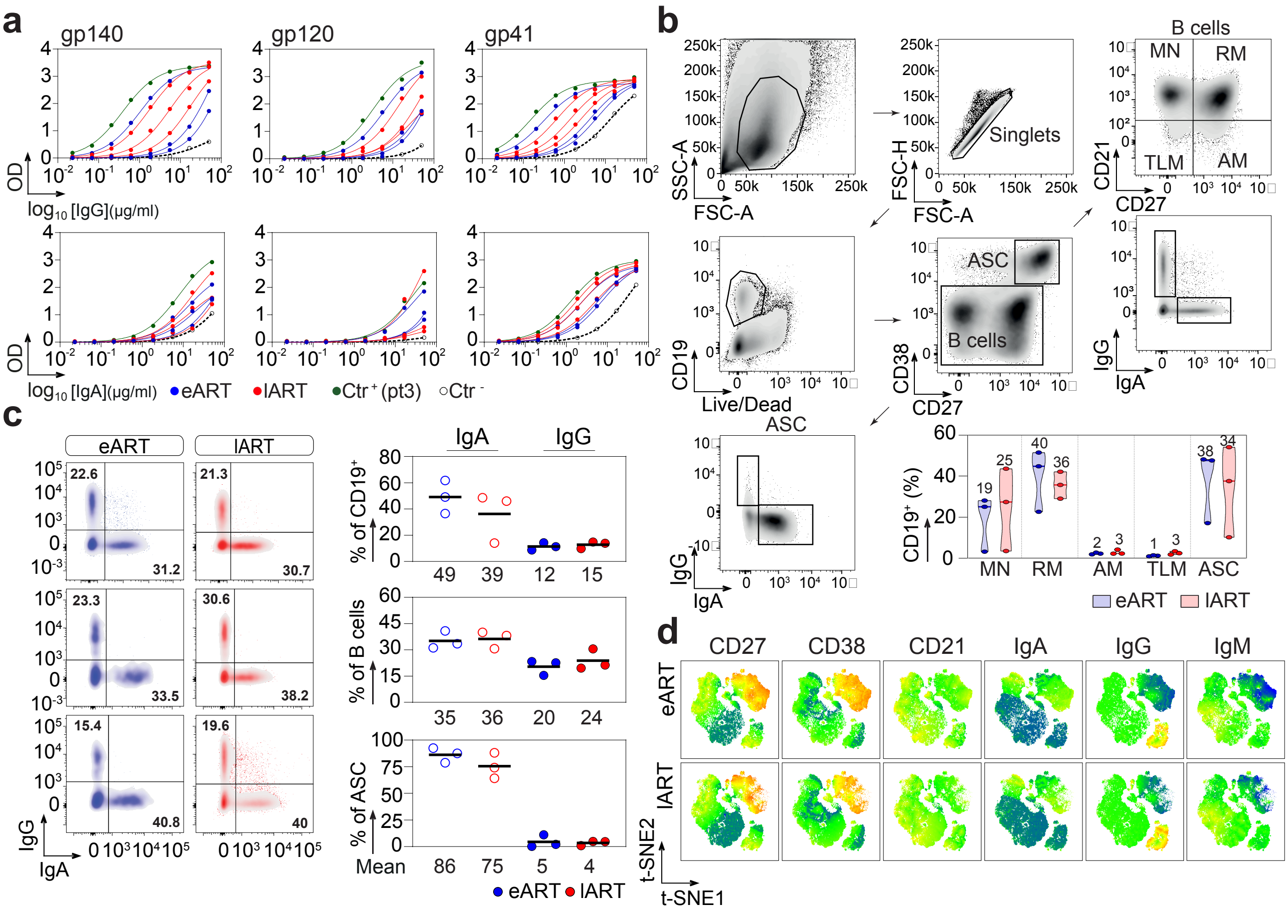

Supplementary Fig. 1 | Intestinal IgA⁺ and IgG⁺ B cells from eART and lART. a ELISA graph showing the reactivity of purified serum IgG and IgA antibodies from eART (n=3, blue) and lART (n=3, red) against trimeric gp140-F, gp120, and gp41 proteins. Means ± SEM of values from two independent experiments (performed in duplicate) are shown. Ctr⁺, positive control (pt3[86]); Ctr⁻, non-HIV-1 negative control. **b** Cytograms (top) and dot plots (bottom) showing the flow cytometry gating strategy and frequency comparison for mature naïve (MN), resting memory (RM), activated memory (AM), tissue-like memory (TLM) B cells and antibody-secreting cells (ASC) between eART (n=3) and lART (n=3). **c** Cytograms (left) showing the flow cytometry gating strategy and dot plots (right) comparing the IgA⁺ and IgG⁺ B-cell frequencies between eART (n=3) and lART (n=3). The average frequency of IgA⁺ and IgG⁺ cells among total CD19⁺ cells, B cells and ASC is indicated below each dot plot. **d** t-SNE-based analysis of intestinal CD19⁺ cells from eART and lART (n=3; 2 x 10^5 cells *per* group). Two-dimensional t-SNE plots depict the expression levels (color-coded: blue (low) to red (high)) of surface IgA, IgG, IgM, CD21, CD38 and CD27 markers. Source data are provided as a Source Data file.

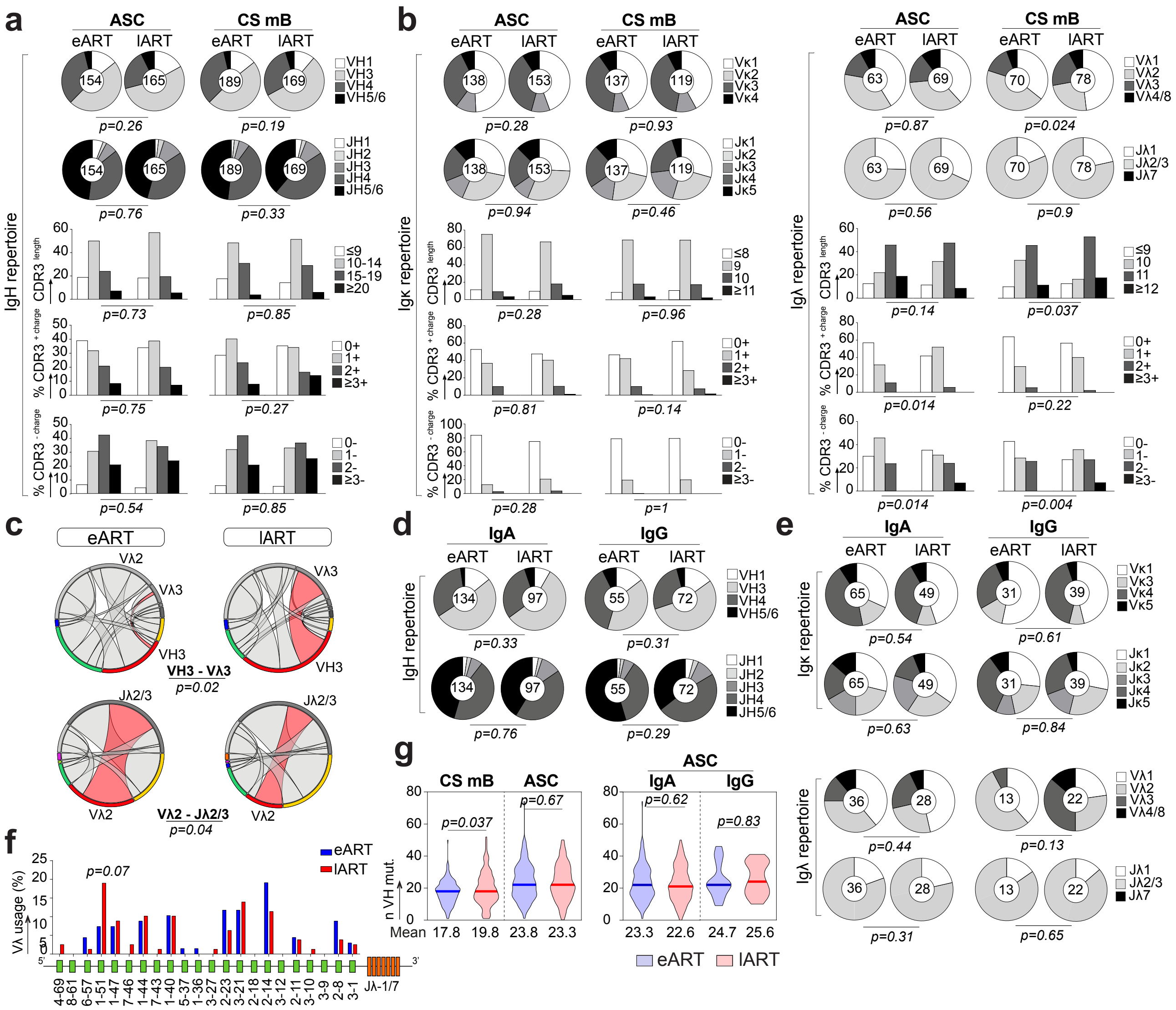

Supplementary Fig. 2 | Immunoglobulin gene features of intestinal IgA⁺ and IgG⁺ B cells. a IgH gene features. Pie charts (top) comparing the distribution of V and J gene usage in mucosal B cells (class-switched memory B cells [CS mB] and ASC) between eART and lART (n=3 *per* group). The number of antibody sequences analyzed is indicated in the center of each pie chart. Groups were compared using 2 × 5 Fisher's Exact test. Bar graphs (bottom) showing the distribution of CDR3 lengths, positive and negative charge numbers in intestinal B-cell antibodies from eART and lART. **b** Same as in (a) but for Igκ (left) and Igλ (right) gene features. **c** Circos plots generated with immunoglobulin gene data of intestinal memory B cells using "circlize" R package (v0.3.1) comparing between eART and lART, VH/Vλ and Vλ/Jλ association and rearrangement frequencies, respectively. Groups were compared using 2 × 5 Fisher's Exact test. **d** Pie charts comparing the distribution of V and J gene usage in IgA⁺ and IgG⁺ memory B cells between eART and lART. **e** Same as in (d) but for Igκ (top) and Igλ (bottom) genes. **f** Bar graphs comparing the Vλ gene distribution in single intestinal memory B cells between eART and lART. Groups were compared using 2 × 2 Fisher's Exact test. **g** Violin plots comparing the number VH gene mutations in gut memory B cells and ASC between eART and lART. The average number of mutations is indicated below each violin plot. Numbers of somatic mutation (n VH mut.) were compared using two-tailed unpaired student t-test with Welch's correction. Source data are provided as a Source Data file.

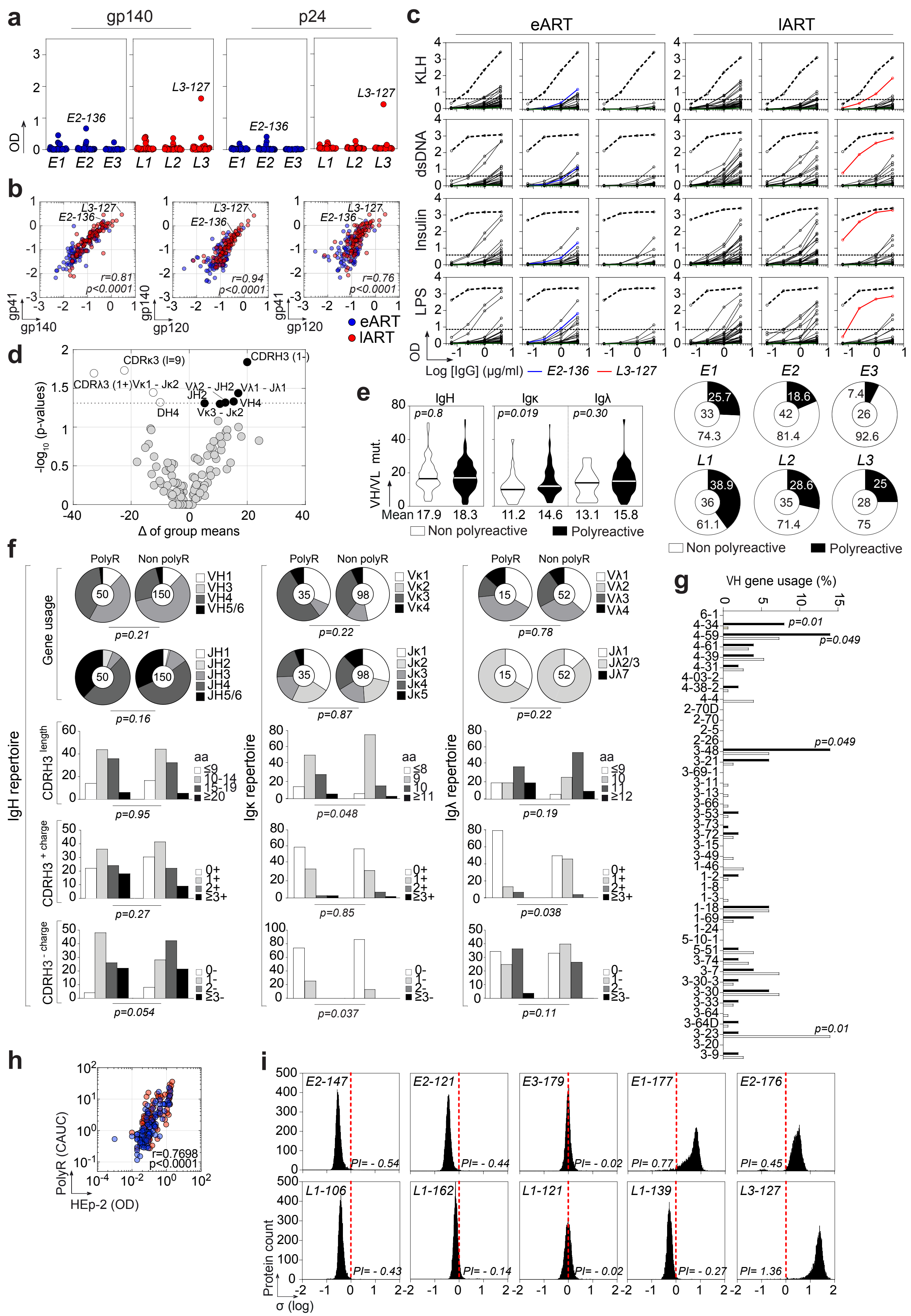
a
gp140
p24
OD
E2-136
L3-127
E1 E2 E3 L1 L2 L3
b
gp41
gp140
gp120
r=0.81 p<0.0001
r=0.94 p<0.0001
r=0.76 p<0.0001
eART
lART
c
eART
lART
KLH
dsDNA
Insulin
LPS
OD
Log [IgG] (μg/ml)
E2-136
L3-127
d
-log10 (p-values)
Δ of group means
CDRκ3 (l=9)
CDRλ3 (1+)
Vκ1 - Jκ2
Vλ2 - JH2
JH2
DH4
CDRH3 (1-)
Vλ1 - Jλ1
VH4
Vκ3 - Jκ2
e
IgH
Igκ
Igλ
p=0.8
p=0.019
p=0.30
VH/VL mut.
Mean 17.9 18.3 11.2 14.6 13.1 15.8
Non polyreactive
Polyreactive
E1 E2 E3
L1 L2 L3
25.7 33 74.3
18.6 42 81.4
7.4 26 92.6
38.9 36 61.1
28.6 35 71.4
25 28 75
Non polyreactive
Polyreactive
f
IgH repertoire
Igκ repertoire
Igλ repertoire
Gene usage
PolyR
Non polyR
VH1 VH3 VH4 VH5/6
JH1 JH2 JH3 JH4 JH5/6
Vκ1 Vκ2 Vκ3 Vκ4
Jκ1 Jκ2 Jκ3 Jκ4 Jκ5
Vλ1 Vλ2 Vλ3 Vλ4
Jλ1 Jλ2/3 Jλ7
p=0.21
p=0.16
p=0.22
p=0.87
p=0.78
p=0.22
CDRH3 length
aa
p=0.95
p=0.048
p=0.19
CDRH3 + charge
p=0.27
p=0.85
p=0.038
CDRH3 - charge
p=0.054
p=0.037
p=0.11
g
VH gene usage (%)
p=0.01
p=0.049
p=0.049
p=0.01
h
PolyR (CAUC)
HEp-2 (OD)
r=0.7698
p<0.0001
i
E2-147 PI= - 0.54
E2-121 PI= - 0.44
E3-179 PI= - 0.02
E1-177 PI= 0.77
E2-176 PI= 0.45
L1-106 PI= - 0.43
L1-162 PI= - 0.14
L1-121 PI= - 0.02
L1-139 PI= - 0.27
L3-127 PI= 1.36
Protein count
σ (log)

Supplementary Fig. 3 | Polyreactivity of intestinal memory B cells. a Dot plots comparing the reactivity of mucosal antibodies (n=200) from eART and lART (n=3 *per* group) against HIV-1 Env proteins (gp140-F, gp120 and gp41) determined in triplicate using classical ELISA. **b** Correlation plots (bottom; $\log_{10}$ OD binding values) comparing the reactivity of mucosal antibodies (n=200) from eART (blue) and lART (red) against HIV-1 Env proteins (gp140-F, gp120 and gp41) using polyreactive ELISA. Bivariate correlations were estimated with the two-tailed Pearson correlation test. **c** ELISA graphs comparing the polyreactivity of the mucosal antibodies cloned from eART and lART donors (n=3 *per* group) against KLH, dsDNA, insulin and LPS. mGO53 (green line) and ED38 (dotted line) are negative and positive control antibodies, respectively. Horizontal lines show cutoff for positive reactivity. Pie charts (below) present the frequency of polyreactive (black) and non-polyreactive (white) intestinal memory B-cell antibodies *per* individual. The number of tested antibodies is indicated in the pie chart center. Means ± SEM of values from two independent experiments (performed with duplicate) are shown. **d** Volcano plots comparing the gene features (n=260 parameters) of polyreactive and non-polyreactive intestinal memory B-cell antibodies. The y axis indicates the statistics expressed as $-\log_{10}$ (p values) and the x axis represents the differences between the group means for each parameter. Dashed lines indicate the significance cut off $p<0.05$. **e** Violin plots comparing the number of VH, Vκ and Vλ gene mutations between poly- and non-polyreactive mucosal antibodies. The average number of somatic mutations is indicated below each violin plot. Numbers of mutation were compared between groups using unpaired student t-test with Welch's correction. **f** IgH, Igκ and Igλ gene features of polyreactive and non-polyreactive antibodies. Pie charts (top) showing the distribution of VH/VL and JH/JL gene usage in polyreactive and non-polyreactive mucosal memory B-cell antibodies. Bar graphs (bottom) showing the distribution of CDR3 lengths, positive and negative charge numbers in polyreactive and non-polyreactive mucosal memory B-cell antibodies. The number of antibody sequences analyzed is indicated in the center of each pie chart. Groups were compared using 2 × 5 Fisher's Exact test. **g** Bar graphs comparing the distribution of VH genes expressed by single poly- and non-polyreactive intestinal memory B-cell antibodies. Groups were compared using 2 × 2 Fisher's Exact test. **h** Correlation plot comparing the levels of antibody polyreactivity (polyR, as cumulative area under the curve [CAUC]) and HEp-2-reactivity determined by ELISA in eART (blue) and lART (red). Bivariate correlation was estimated using the two-tailed Pearson correlation test. **i** Frequency histograms show the $\log_{10}$ protein displacement (σ) of the mean fluorescent intensity (MFI) signals for the selected antibodies (n = 10) compared with non-reactive antibody mGO53. The polyreactivity index (PI) corresponds to the Gaussian mean of all array protein displacements. Source data are provided as a Source Data file.

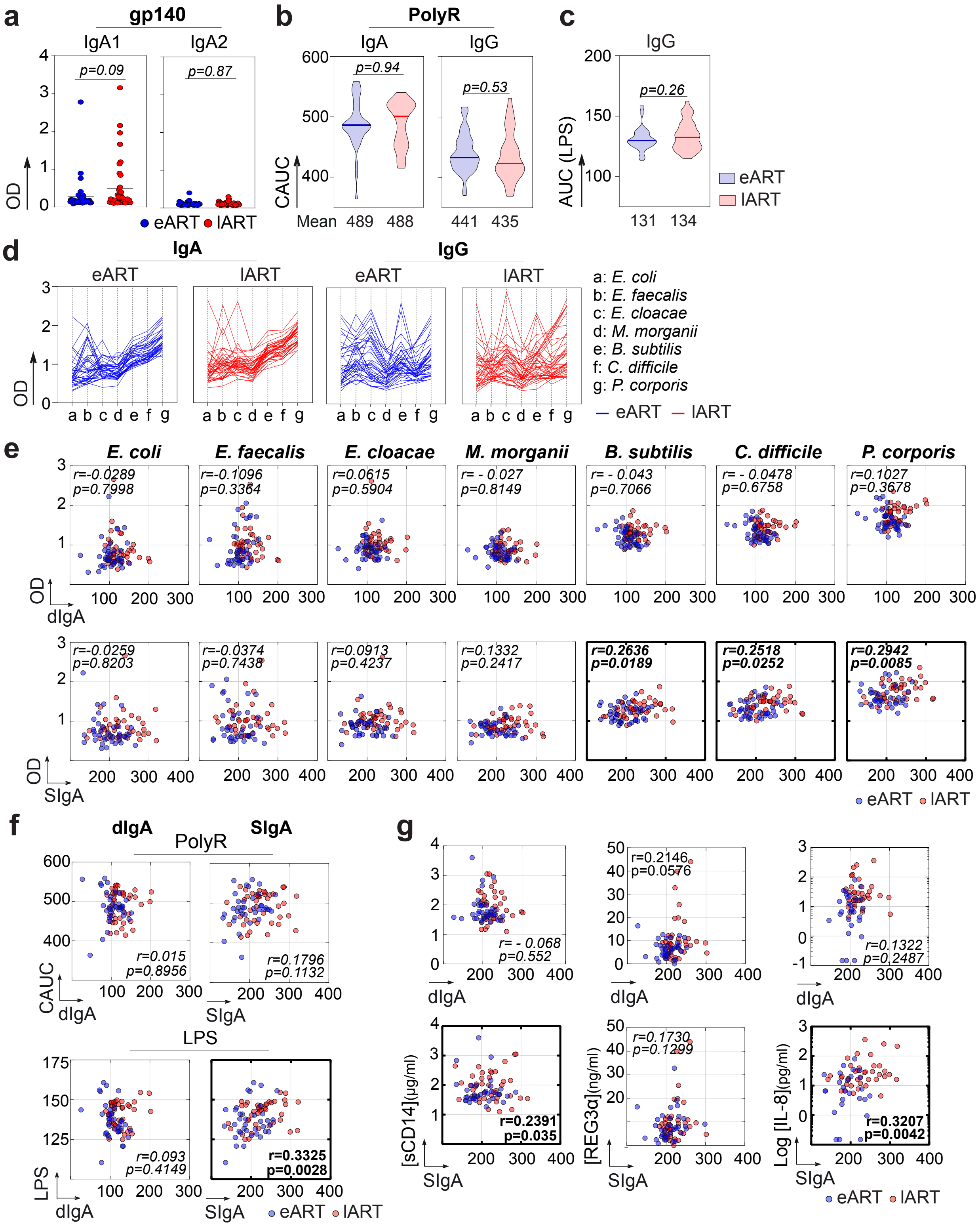

Supplementary Fig. 4 | Reactivity of serum IgA and IgG antibodies from eART and lART. **a** Dot plots comparing the IgA1 and IgA2 reactivity against HIV-1 gp140-F of purified serum IgA antibodies between eART (n=38) and lART (n=40). Each dot corresponds to the mean of triplicate values. Groups were compared using Student's t-test with Welch's correction. **b** Violin plots comparing the polyreactivity of purified serum IgA and IgG antibodies from eART (n=38) and lART (n=40). Mean values from two independent experiments (performed in duplicate) were used. The y axis indicates the CAUC values for the polyreactivity. CAUC mean is indicated below each violin plot. **c** Violin plots comparing the reactivity of purified IgG antibodies against LPS. Mean values from two independent experiments (performed in duplicate) were used.The y axis indicates the AUC values for the LPS reactivity. AUC mean is indicated below each violin plot. **d** Graphs comparing the reactivity profiles against commensal bacteria of purified serum IgA and IgG antibodies from eART (n=38) and lART (n=40). Means from duplicate values are shown. **e** Correlation plots comparing total serum dIgA and SIgA levels with the IgA reactivity against selected commensal bacteria in eART eART (n=38) and lART (n=40). **f** Correlation plots comparing total serum dIgA and SIgA levels with the IgA poly- and LPS-reactivity in eART and lART. **g** Correlation plots comparing serum dIgA and SIgA levels with the serum concentration of sCD14, REG3α and IL-8. Bivariate correlations were estimated with the two-tailed Pearson correlation test.with Welch's correction. Source data are provided as a Source Data file.

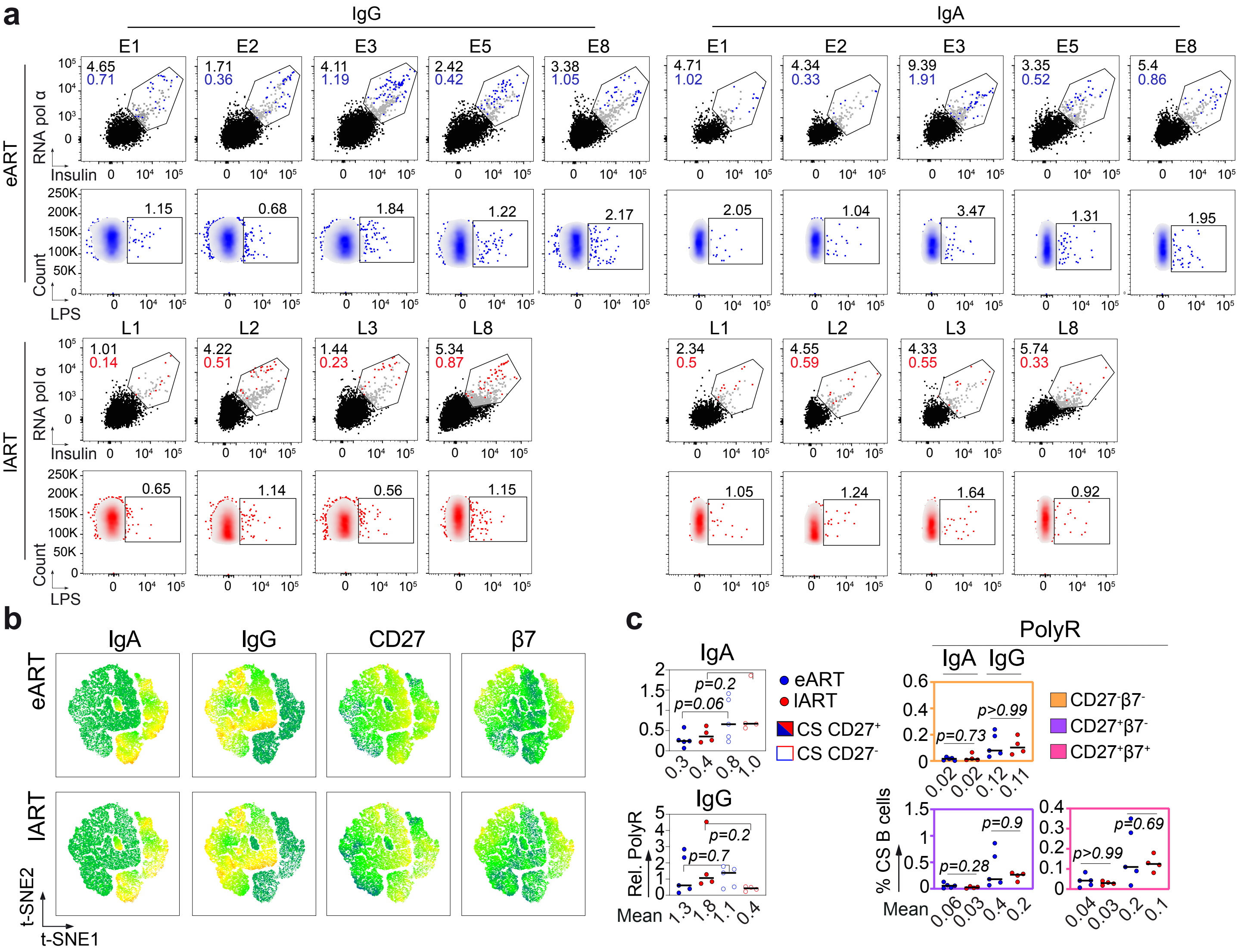

Supplementary Fig. 5 | Polyreactive binding of class-switched memory B cells from eART and lART. a Flow cytograms showing the gating strategy used to determine the frequency of polyreactive blood IgA$^+$ and IgG$^+$ B cells from eART (n=5) and lART (n=4). The frequency of antigen-reactive cells is indicated above each gate. Colored values indicate the frequency of B cells reactive with all antigens tested (RNApol α, LPS and Insulin), which were defined as polyreactive (polyR) B cells. **b** t-SNE-based analysis of blood IgA$^+$ and IgG$^+$ B cells from eART (n=4) and lART (n=5) (2 x 10^5 cells *per* group). Two-dimensional t-SNE plots depict the expression levels (color-coded: blue (low) to red (high)) of IgA, IgG, CD27 and β7 surface markers. **c** Dot plots comparing the relative polyreactivity (rel. PolyR; calculated as the % of polyR B cells / % of total B cells in the subset) of CD27$^+$ (plain circles) or CD27$^-$ (open circles) class-switched (CS) B-cell sub-populations between eART (blue) and lART (red) (left). The average value is indicated below each dot plot. Dot plots comparing between eART and lART the distribution of polyreactive cells (PolyR) among the B-cell subsets described in Fig. 5b. The average frequency of cells among the total IgA$^+$ and IgG$^+$ B cells is indicated below each dot plot. Groups were compared using Student's t-test with Welch's correction. Source data are provided as a Source Data file.
