# Supplementary material for: HIV-1 treatment timing shapes the human intestinal memory B-cell repertoire to commensal bacteria
Source: Nat Commun. 2023 Oct 10;14:6326. doi: 10.1038/s41467-023-42027-6 (PMC10564866; doi:10.1038/s41467-023-42027-6)
Supplement: Supplementary file 3 — Reporting Summary [file 41467_2023_42027_MOESM3_ESM.pdf]

nature portfolio

Corresponding author(s): Hugo MOUQUET

Last updated by author(s): Sep 8, 2023

Reporting Summary

Nature Portfolio wishes to improve the reproducibility of the work that we publish. This form provides structure for consistency and transparency in reporting. For further information on Nature Portfolio policies, see our Editorial Policies and the Editorial Policy Checklist.

Statistics

For all statistical analyses, confirm that the following items are present in the figure legend, table legend, main text, or Methods section.

n/a	Confirmed	
☐	☒	The exact sample size (*n*) for each experimental group/condition, given as a discrete number and unit of measurement
☐	☒	A statement on whether measurements were taken from distinct samples or whether the same sample was measured repeatedly
☐	☒	The statistical test(s) used AND whether they are one- or two-sided<br>*Only common tests should be described solely by name; describe more complex techniques in the Methods section.*
☐	☒	A description of all covariates tested
☐	☒	A description of any assumptions or corrections, such as tests of normality and adjustment for multiple comparisons
☐	☒	A full description of the statistical parameters including central tendency (e.g. means) or other basic estimates (e.g. regression coefficient) AND variation (e.g. standard deviation) or associated estimates of uncertainty (e.g. confidence intervals)
☐	☒	For null hypothesis testing, the test statistic (e.g. *F*, *t*, *r*) with confidence intervals, effect sizes, degrees of freedom and *P* value noted<br>*Give P values as exact values whenever suitable.*
☒	☐	For Bayesian analysis, information on the choice of priors and Markov chain Monte Carlo settings
☒	☐	For hierarchical and complex designs, identification of the appropriate level for tests and full reporting of outcomes
☐	☒	Estimates of effect sizes (e.g. Cohen's *d*, Pearson's *r*), indicating how they were calculated

Our web collection on statistics for biologists contains articles on many of the points above.

Software and code

Policy information about availability of computer code

Data collection	ELISA: Sunrise™ microplate absorbance reader (Tecan Männedorf, Switzerland). Magellan software v7.2 (TECAN)<br>IFA: Axio Imager 2 (Zeiss) fluorescence microscope. ZEN imaging software (Zen 2.0 blue version, Zeiss)<br>Dot-blotting: Odyssey Infrared Imaging system (LI-COR Biosciences).<br>Neutralization: Wallac 1420 VICTOR2™ microplate reader (Perkin Elmer). Wallac 1420 Workstation<br>Flow cytometry for B-cell immunophenotyping: FACS LSRII (Becton Dickinson). FACS Diva v8.0.1 (BD)<br>Single-cell sorting: BDFACSAria II™ instrument (BD Biosciences). FACS Diva v8.0.1 (BD)<br>Protein microarray: GenePix 4000B microarray scanner (Molecular Devices)<br>High-throughput immunoglobulin sequencing: Miseq instrument (Illumina)
Data analysis	GraphPad Prism v8.0.0 to v10.0.0 (GraphPad)<br>R Studio Server (v1.4.3). FactoMineR package (v2.4, https://CRAN.R-project.org/package=FactoMineR). Factoextra package (v1.0.7, https://CRAN.R-project.org/package=factoextra). Hmisc package (v4.7, https://CRAN.R-project.org/package=Hmisc). Corrplot package (v0.92, https://CRAN.R-project.org/package=corrplot)<br>FlowJo (v10.7.1, FlowJo LLC, Ashland, OR)<br>Qlucore Omics Explorer software v3.7 (Qlucore AB)<br>ZEN imaging software (Zen 2.0 blue version, Zeiss)<br>GenePix Pro 6.0 software (Molecular Devices)<br>Spotxel® software (SICASYS Software GmbH)<br>ProtoArray® Prospector software (v5.2.3, Thermo Fisher Scientific)<br>PEAR software

IgBlast (https://www.ncbi.nlm.nih.gov/igblast/)
CD-HIT (http://weizhongli-lab.org/cd-hit/)

For manuscripts utilizing custom algorithms or software that are central to the research but not yet described in published literature, software must be made available to editors and reviewers. We strongly encourage code deposition in a community repository (e.g. GitHub). See the Nature Portfolio guidelines for submitting code & software for further information.

Data

Policy information about availability of data

All manuscripts must include a data availability statement. This statement should provide the following information, where applicable:

- Accession codes, unique identifiers, or web links for publicly available datasets
- A description of any restrictions on data availability
- For clinical datasets or third party data, please ensure that the statement adheres to our policy

Source data are provided in the manuscript.
All statistical values calculated from the multiparameter correlations are also provided.
Raw data or information on immunoglobulin gene sequences from single B cells and Ig-HTS are available upon reasonable request to the corresponding author.

Research involving human participants, their data, or biological material

Policy information about studies with human participants or human data. See also policy information about sex, gender (identity/presentation), and sexual orientation and race, ethnicity and racism.

Reporting on sex and gender	The sex/gender of all human participants is indicated in the supplemental table S1, and was not considered in the study design nor in data analysis.
Reporting on race, ethnicity, or other socially relevant groupings	No reports were performed on ethnicity, or other socially relevant groupings.
Population characteristics	Biological samples where obtained from HIV-1-infected individuals under effective antiretroviral therapy (ART) for several years at the Centre Hospitalier Regional d'Orléans. ART was started within 4 months after the diagnosis of primary HIV-1 infection (Fiebig stage II-V) in 38 patients (eART; median age 48 [28-79] years) or during the chronic stage of HIV-1 infection (Fiebig stage VI) in 40 patients (lART; median age 50 [23-80] years). The main clinical and immuno-virological characteristics of the study participants are provided in the Supplementary Table 1.
Recruitment	Participants in the study were randomly selected but, based on ART timing either during primary HIV-1 infection (Fiebig stage II-V) - eART goup - or the chronic stage of infection - lART group. Median age-match checking between groups was verified after the selection.
Ethics oversight	Samples were obtained as part of the research protocol called BHUANTIVIH performed in accordance with and after ethical approval from all the French legislation and regulation authorities. All donors gave written consent to participate in this study, and data were collected under pseudo-anonymized conditions using subject coding. The clinical research protocol received approval from the Comité Consultatif pour le Traitement de l'Information en matière de Recherche dans le domaine de la Santé (CCTIRS) on December 12th 2013, the Commission Nationale de l'Informatique et des Libertés (CNIL) on August 8th 2014 and the Comité de Protection des Personnes de Tours (CPP Région Centre-Ouest 1) on December 17th 2014. Ethical issues have been monitored by the Ethics Board for European contracts, an ad hoc independent Ethics Committee in charge of reviewing periodically sensitive ethical issues in EU funded research when requested by the EU.

Note that full information on the approval of the study protocol must also be provided in the manuscript.

Field-specific reporting

Please select the one below that is the best fit for your research. If you are not sure, read the appropriate sections before making your selection.

☒ Life sciences ☐ Behavioural & social sciences ☐ Ecological, evolutionary & environmental sciences

For a reference copy of the document with all sections, see nature.com/documents/nr-reporting-summary-flat.pdf

Life sciences study design

All studies must disclose on these points even when the disclosure is negative.

Sample size	This is a descriptive and exploratory study on difficult-to-obtain gut samples from HIV-infected individuals. Sample size determination was therefore not applied; we included and analyzed all available tissue biopsies obtained during the study (with matched blood cell samples).<br>Each antibody was assumed to be statistically independent; about 30 antibodies per donor with 3 donors per group were analyzed (Average n=34[26-42] for eART and n=33[28-36] for lART).<br>For the analyses on patients' sera, we tested all bio-banked sample available in the lab (38 from eART and 40 from lART).
Data exclusions	No data exclusions were performed.

Replication	All ELISA binding experiments were performed in duplicate or triplicate, in at least two independent experiments.<br>Indirect immunofluorescence assays were also performed twice in two different experiments.<br>All experiments were reproduced successfully.<br>Protein microarray binding analyses and flow cytometry immunophenotyping data were obtained in a single experiment.<br>Testing of purified serum IgG antibodies in the in vitro HIV-1 neutralization assay was performed once in triplicate.
Randomization	Randomization of the study populations does not fully apply; the donors were randomly-selected but first based on defined treatment timing - either eART or lART.
Blinding	Blinding was not applied because: (i) this was not an interventional study, and (ii) individuals were selected according to a defined criterium (see aforementioned).

Reporting for specific materials, systems and methods

We require information from authors about some types of materials, experimental systems and methods used in many studies. Here, indicate whether each material, system or method listed is relevant to your study. If you are not sure if a list item applies to your research, read the appropriate section before selecting a response.

Materials & experimental systems

n/a	Involved in the study
☐	☒ Antibodies
☐	☒ Eukaryotic cell lines
☒	☐ Palaeontology and archaeology
☒	☐ Animals and other organisms
☒	☐ Clinical data
☒	☐ Dual use research of concern
☒	☐ Plants

Methods

n/a	Involved in the study
☒	☐ ChIP-seq
☐	☒ Flow cytometry
☒	☐ MRI-based neuroimaging

Antibodies

Antibodies used	ELISAs - Secondary conjugated antibodies used for revelation: Polyclonal goat peroxydase-conjugated anti-human IgG (#109-035-098) and anti-human IgA (#109-035-011) [Immunology Jackson ImmunoReseach]. The antibodies were used at a final concentration of 0.8 µg/ml. High-binding 96-well ELISA plates (Costar, Corning) were also coated overnight with 250 ng/well of<br>mouse anti-human J chain (Mc19-9, #MCA693, BIO-RAD) and anti-human secretory component (GA-1, #I6635, Sigma-Aldrich) antibodies. mGO53 (PMID: 12920303) and ED38 (PMID: 14699083) are human recombinant monoclonal antibodies used as negative and positive controls, respectively. These two antibodies were used at different concentrations depending on the experimental design.<br>Flow cytometry - B-cell antibody panel: CD19 A700 (HIB19, #557921, 1:100 dilution), CD38 APC (HIT2, #560980, BD Biosciences, 1:100 dilution), CD21 BV421 (B-ly45, #562966, 1:100 dilution), CD27 PE-CF594 (M-T271, #562297, BD Biosciences, 1:100 dilution), CD27 BV711 (M-T271, #564893, 1:100 dilution), IgM BV605 (G20-127, #562977, BD Biosciences, 1:100), IgG BV605 (G18-145, #563246, BD Biosciences, 1:100), IgG BV786 (G18-145, #564230, BD Biosciences, 1:100), IgA FITC (IS11-8E10, #130.114.001, 1:100 dilution, Miltenyi Biotec) and Integrin β7 BUV395 (FIB504, #744014, BD Biosciences,1:100 dilution).<br>Protein microarray binding analysis - Secondary conjugated antibodies used for revelation: The polyclonal goat anti-Human IgG (H+L) Cross-Adsorbed Secondary Antibody, Alexa Fluor™ 647 (# A-21445, ThermoFischer) was used at 1 µg/ml.
Validation	Most of the antibodies are commercially available and the reactivity validated against the appropriate species, as reported in manufactuer´s website, technical datasheets and previous studies.<br><br>Mouse anti-human CD19 A700 antibody (clone HIB19, #557921, BD Biosciences): According to manufacturer´s website the antibody is routinely tested in flow cytometry. Flow cytometric analysis of CD19 expression on human peripheral blood lymphocytes is provided in the website and technical datasheet. Supporting references are also provided.<br><br>Mouse anti- human CD38 APC antibody (clone HIT2, #560980, BD Biosciences): According to manufacturer´s website the antibody is routinely tested in flow cytometry. Flow cytometric analysis of CD38 expression on human peripheral blood lymphocytes is provided in the website and technical datasheet. Supporting references are also provided.<br><br>Mouse anti-human CD21 BV421 antibody (clone B-ly45, #562966, BD Biosciences): According to manufacturer´s website the antibody is routinely tested in flow cytometry. Flow cytometric analysis of CD21 expression on human peripheral blood lymphocytes is provided in the website and technical datasheet. Supporting references are also provided.<br><br>Mouse anti-human CD27 PE-CF594 antibody (clone M-T271, #562297, BD Biosciences): According to manufacturer´s website the antibody is routinely tested in flow cytometry. Flow cytometric analysis of CD27 expression on human peripheral blood lymphocytes is provided in the website and technical datasheet. Supporting references are also provided.<br><br>Mouse anti-human CD27 BV711 antibody (clone M-T271, #564893, BD Biosciences): According to manufacturer´s website the antibody is routinely tested in flow cytometry. Flow cytometric analysis of CD27 expression on human peripheral blood lymphocytes

is provided in the website and technical datasheet. Supporting references are also provided.

Mouse anti-human IgM BV605 antibody (clone G20-127, #562977, BD Biosciences): According to manufacturer´s website the antibody is routinely tested in flow cytometry. A two color flow cytometric analysis of IgM expression on human peripheral blood lymphocytes is provided in the website and technical datasheet. Supporting references are also provided.

Mouse anti-human IgG BV605 antibody (clone G18-145, #563246, BD Biosciences): According to manufacturer´s website the antibody is routinely tested in flow cytometry. A two color flow cytometric analysis of IgG expression on human peripheral blood lymphocytes is provided in the website and technical datasheet. Supporting references are also provided.

Mouse anti-human IgG BV786 antibody (clone G18-145, #564230, BD Biosciences): According to manufacturer´s website the antibody is routinely tested in flow cytometry. A two color flow cytometric analysis of IgG expression on human peripheral blood lymphocytes is provided in the website and technical datasheet. Supporting references are also provided.

Mouse anti-human IgA FITC antibody (clone IS11-8E10, #130-114-001, Miltenyi Biotec): According to manufacturer´s website, the antibody is suited for flow cytometry. Flow cytometric analysis of IgA expression on human peripheral blood mononuclear cells is provided in the website and technical datasheet. Extended validations on epitope specificity, sensitivity and fixation data are also provided. Supporting references are also provided.

Rat anti-human Integrin β7 BUV395 antibody (clone FIB504, #744014, BD Biosciences): According to manufacturer´s website the antibody is qualified for flow cytometry. Supporting references are provided.

Mouse anti-human J chain antibody (clone Mc19-9, # MCA693, BIO-RAD): According to manufacturer´s website the antibody is qualified for ELISA binding experiments and Western blotting. ELISA binding analysis was performed on purified human Ig fragments as the solid phase target antigen. Supporting references are provided.

Mouse anti-human secretory component (IgA) antibody (clone GA-1, #I6635, Sigma-Aldrich): According to manufacturer´s website the antibody is qualified for ELISA binding experiments. ELISA binding analysis was performed on secretory component purified from human colostrum. Supporting references are provided.

mGO53 (PMID: 12920303) and ED38 (PMID: 14699083) are human recombinant monoclonal antibodies used as negative and positive controls, respectively.

The polyclonal goat anti-Human IgG (H+L) Cross-Adsorbed Secondary Antibody, Alexa Fluor™ 647 (# A-21445, ThermoFischer): Antibody specificity was demonstrated by detection of differential basal expression of IgG across cell lines owing to their inherent genetic constitution. Relative expression of Human IgG was observed in IM-9, ARH-77 and IM-9, ARH-77 conditioned medium (CM) but not in Raji, MOLT-4, Jurkat and Jurkat CM using Goat anti-Human IgG (H+L) Cross-Adsorbed Secondary Antibody, Alexa Fluor 647 (Product # A- 21445) in Western Blot. IM-9 and ARH-77 express and secrete IgG whereas Raji is known to express IgM. MOLT-4 and Jurkat (T-cell lines) do not express immunoglobulins. Supporting references are provided.

Eukaryotic cell lines

Policy information about cell lines and Sex and Gender in Research

Cell line source(s)	HEK-293T cells (CRL-11268™, ATCC).<br>Freestyle™ 293-F cells (R79007, Thermo Fisher Scientific).<br>TZM-bl cells (#8129, NIH AIDS Reagent Program).
Authentication	Authentication was based on morphology, growth and expected behaviour and functionality.
Mycoplasma contamination	Mycoplasma contamination not tested.
Commonly misidentified lines<br>(See ICLAC register)	Misidentified lines were not used.

Flow Cytometry

Plots

Confirm that:

- [x] The axis labels state the marker and fluorochrome used (e.g. CD4-FITC).
- [x] The axis scales are clearly visible. Include numbers along axes only for bottom left plot of group (a 'group' is an analysis of identical markers).
- [x] All plots are contour plots with outliers or pseudocolor plots.
- [x] A numerical value for number of cells or percentage (with statistics) is provided.

Methodology

Sample preparation	Single B-cell flow cytometry phenotyping, sorting and expression-cloning of antibodies: Intraepithelial lymphocytes (IEL) were isolated by two rounds of vigorous shaking in DMEM-Glutamax (Gibco, Thermo Fisher Scientific) supplemented with 1% Fetal

Bovine Serum (FBS) (Gibco), 1 mM EGTA and 1.5 mM MgCl2. Lamina propria lymphocytes (LPL) were isolated by two rounds of tissue digestion in medium containing collagenase II 100U/ml (Sigma), followed by mechanical disruption with a syringe equipped with a 16-gauge blunt-end needle. IEL and LPL were pooled and washed in DMEM - Glutamax (Gibco) supplemented with 10% FBS (Gibco), 1% penicillin and 1% streptomycin (Gibco). Purified mucosal cells were first stained using LIVE/DEAD aqua fixable dead cell stain kit (Molecular Probes, Thermo Fisher Scientific) to exclude dead cells. Cells were then incubated for 30 min at 4°C with biotinylated YU2 gp140-F trimers, washed once with 1% FBS-PBS (FACS buffer), and incubated for 30 min at 4°C with a cocktail of mouse anti-human antibodies: CD19 Alexa 700 (HIB19, BD Biosciences), CD38 APC (HIT2, BD Biosciences), CD21 BV421 (B-ly4, BD Biosciences), CD27 PE-CF594 (M-T271, BD Biosciences), IgM BV605 (G20-127, BD Biosciences), IgG BV786 (G18-145, BD Biosciences), IgA FITC (IS11-8E10, Miltenyi Biotec), Integrin β7 BUV395 (FIB504, BD Biosciences) and streptavidin R-PE conjugate (Invitrogen, Thermo Fisher Scientific). Cells were then washed and resuspended in FACS buffer.

Flow cytometry B-cell binding assay: PBMC were isolated from donors' blood using Ficoll Plaque Plus (GE Healthcare). PBMC were first stained using LIVE/DEAD aqua fixable dead cell stain kit (Molecular Probes, Thermo Fisher Scientific) to exclude dead cells. Cells were then incubated for 30 min at 4°C with biotinylated HIV-1 gp140 trimer, DyLight 650-coupled Insulin, DyLight 405-coupled RNA polymerase α and Alexa Fluor 594-coupled LPS (ThermoFisher scientific), washed once with 1% FBS-PBS (FACS buffer), and incubated for 30 min at 4°C with a cocktail of mouse anti-human antibodies: CD19 Alexa 700 (HIB19, BD Biosciences, San Jose, CA), CD21 BV421 (B-ly4, BD Biosciences), CD27 BV711 (M-T271, BD Biosciences), IgG BV605 (G18-145, BD Biosciences), IgA FITC (IS11-8E10, Miltenyi Biotec, Bergisch Gladbach, Germany), Integrin β7 BUV395 (FIB504, BD Biosciences) and streptavidin R-PE conjugate (Thermo Fisher Scientific). Cells were then washed and resuspended in FACS buffer.

Instrument

- BD LSRFortessa™ instrument (BD Biosciences)
- BDFACSAria II™ instrument (BD Biosciences).

Software

FlowJo software (v10.7.1, FlowJo LLC)

Cell population abundance

N/A

Gating strategy

Lymphocytes were gated based on FSC-A/SSC-A, followed by single cell check based on SSC-A/SSC-H. Live cells were then gated based on a LIVE/DEAD fixable dead cell stain kit (405 nm excitation, Molecular Probes, ThermoFicher Scientific). B-cell subsets were then analyzed using the specified antibody cocktails following the gating strategies shown in Supplementary Figure 1b.

☒ Tick this box to confirm that a figure exemplifying the gating strategy is provided in the Supplementary Information.
